# Sodium Butyrate Ameliorated Bile Acid Metabolism in Diabetes Mellitus by PI3K/AKT Signaling Pathway via the Gut–Liver Axis

**DOI:** 10.3390/cimb47090732

**Published:** 2025-09-09

**Authors:** Tingting Zhao, Xi Zhang, Qian Xiang, Yadi Liu, Xuling Li, Junling Gu, Wenqian Zhang, Zhe Wang, Yiran Li, Xiaoshan Lai, Yonghua Zhao, Youhua Xu

**Affiliations:** 1 Faculty of Chinese Medicine, Macau University of Science and Technology, Taipa, Macao 999078, China; zhaotingting199309@163.com (T.Z.); zhangxi1898@163.com (X.Z.); 17765704572@163.com (Q.X.); liuyadi20190627@163.com (Y.L.); 13760382232@163.com (X.L.); gjl810615@foxmail.com (J.G.); zhangwenqian93@126.com (W.Z.); 18663087018@163.com (Z.W.); m15528032110@163.com (Y.L.); irisin0420@gmail.com (X.L.); 2 State Key Laboratory of Quality Research in Chinese Medicine, Institute of Chinese Medical Sciences, University of Macau, Macao 999078, China; yonghuazhao@um.edu.mo; 3 Zhuhai MUST Science and Technology Research Institute, Macau University of Science and Technology, Hengqin, Zhuhai 519099, China

**Keywords:** diabetes, sodium butyrate, bile acid, mitochondria, gut–liver axis

## Abstract

The liver and gut play a central role in modulating bile acid metabolism. Our recent study found that supplementation with sodium butyrate (NaB) from microbiota might slow diabetes progression and ameliorate liver function in diabetic mice. The role of NaB in the homeostasis of mitochondrial energy metabolism and bile acid metabolism needs to be investigated further, so this study was conducted by us. We used an ELISA kit to detect biochemical indicators related to mice; HE and PAS were used to stain and analyze tissues; CCK8 was used to detect cell viability; and WB was used to detect related indicators. We found here that NaB administration enormously reduced liver hypertrophy and steatosis in diabetic mice, improved liver and gut function and the release of inflammatory factors in diabetic mice, and ameliorated mitochondrial function both in vitro and in vivo. NaB incubation significantly increased bile acid metabolism-related receptors under diabetic conditions; the intracellular content of enzymes related to liver function was elevated within liver cells. Glucose transport proteins GLUT2 and NaB receptor GPR43 were upregulated by NaB on the cell membrane. The actuation of the intracellular signaling proteins PI3K, AKT, and GSK3 was inhibited by NaB under diabetic conditions. The present study proved that the microbiota metabolite NaB has positive effects on bile acid metabolic homeostasis by promoting mitochondrial energy metabolism in enterocytes and the liver, and the GPR43-PI3K-AKT-GSK3 signaling pathway should contribute to this effect.

## 1. Introduction

Type 2 diabetes mellitus (T2DM) is an endocrine and metabolic disease that manifests as insulin resistance and impaired pancreatic β-cell function [1,2]. The main symptom of diabetes is hyperglycemia. Under the long-term influence of hyperglycemia, the body of T2DM patients sustains damage to various tissues and organs, leading to chronic complications such as diabetic retinopathy, diabetic nephropathy and blindness, diabetic neuropathy, and diabetic foot [3,4]. Although the etiology of T2DM is still not fully understood, recent studies have shown that the occurrence and development of T2DM is closely related to the disorder of intestinal flora metabolites [5].

The intestinal flora is an important part of the human body; it plays an important role in improving human energy metabolism and influencing the inflammatory response, and its metabolites are closely connected to human health [6,7]. It was found that the metabolites of the normal intestinal flora within the intestinal lumen can regulate energy metabolism, maintain stability of the internal environment, and improve the body’s immunity, while metabolites from the disordered intestinal flora may promote the occurrence and development of T2DM [8]. The incidence of T2DM is increasing worldwide, and dysbiosis of the gut microbiota may contribute to the development of this disease. Gut microbiota-derived metabolites, including bile acids (BAs), lipopolysaccharides, tryptophan and indole derivatives, and short-chain fatty acids (SCFAs), have been shown to be involved in the pathogenesis of T2DM [9,10]. Studies have found that T2DM patients have changes in the intestinal flora in that the number and proportion of intestinal *Firmicutes*, such as *Lactobacillus*, *Enterococcus*, and the *Firmicutes* belonging to the phyla, was increased, while Bacteroides belonging to the phyla *Bacteroidetes* and *Bifidobacteria* and Rosella belonging to the phyla *Actinobacteria* was decreased. This variation may change patterns of glucose and lipid metabolism, cause inflammation, and induce insulin resistance, finally leading to the occurrence of T2DM [11,12]. Accordingly, a series of theories based on intestinal dysbacteriosis are suggested, such as endotoxin theory is the idea that lipopolysaccharide (LPS) endotoxins contribute to the pathogenesis of T2DM disorder. SCFAs theory is the idea that SCFAs are potential method by which intestinal flora contributes to the establishment and progression of T2DM [13]. For example, low-molecular-weight Laminaria japonica polysaccharide (LJO) can improve the composition of intestinal microbiota and improve the concentration of SCFAs in T2DM mice, and LJO may be a potential dietary supplement for T2DM patients [14].

Butyric acid is one of the metabolites of intestinal flora and is the principal energy source of colon and cecal epithelial cells. It plays a key role in promoting cell differentiation and maturation, maintaining the homeostasis of the intestinal environment, and regulating gene expression [15]. Further studies indicated that butyrate administration can prevent and ameliorate the development of T2DM by promoting energy expenditure and improving mitochondrial functions [16], and G protein-coupled receptor 43 (GPR43) and glucose transporter 2 (GLUT-2) may play a role in this process [17]. As has been well demonstrated, BAs plays a pivotal role in regulating the metabolism of cholesterol and triglycerides [18], and the intestine–liver axis mediates the balance between intestinal bacteria and bile acid metabolism [19]. For example, the anti-T2DM effect of glycodeoxycholic acid is related to the regulation of BAs and gut microbiota composition [20]. To investigate the role of butyrate in intestine–liver axis-mediated BA metabolism, the present study was carried out.

## 2. Materials and Methods

### 2.1. Materials

HepG2 cells and CaCo_2_ cells were kindly provided by the research group of Professor Zhang Wei from the State Key Laboratory of Macau University of Science and Technology. Sodium butyrate (NaB) was obtained from Meilun Biological Technology (Dalian, China). Insulin was provided by Sigma in the United States. Primary antibodies against GPR43 (bs-13536R), PTPN6 (bs-4158R), GATA4 (bs-1778R), GLUT2 (bs-0351R), Insulin Receptor (bs-0681R), PI3K (bs-10657R), and p-PI3K (bs-6417R) were obtained from Bioss (Peking, China). The TGR5 (ab72608) primary antibody was purchased from Abcam (Cambridge, UK). The β-actin primary antibody (AF5001) was obtained from the Beyotime Company (Shanghai, China). Antibodies against AKT (sc-5298), FXR (sc-25309), p-AKT (sc-293125), CYP7A1 (sc-518007), GSK3α/β (sc-7291), and p-GSK3α/β (sc-81496) were purchased from Santa Cruz Biotechnology (Dallas, TX, USA). Commercially available materials and reagents are used for the rest of the experiment. MitoTracker™ Deep Red FM (M22426) were purchased from Thermo Fisher Scientific (Waltham, MA, USA).

### 2.2. Animal Experiment

NaB dosage selection: Our NaB dosage was determined based on our previous research [21], with the final dosage being 0.5 g/kg/day.

According to the conversion formula: mice dosage (g/kg)/0.11 (conversion coefficient) = human dosage (g/kg), This means a 70 kg adult consumes 38.5 g of sodium butyrate per day, which is similar to the recommended daily intake for patients. This suggests that our dose selection is relevant to the patients’ recommended daily intake.

Research on animals followed the Guidelines for the Care and Use of Laboratory Animals published by the animal welfare bodies and national committees [22] and was approved by Macau University of Science and Technology (MUST). Male C57BL/6J mice were provided by Guangdong Medical Laboratory Animal Center. All mice were kept in the animal room with a daytime temperature of 25 °C and a cycle of 12 h. Prior to drug intervention, all of the mice were adaptively fed for 1 week. The model group animals were fed a high-fat diet (43% carbohydrate, 15% protein, 42% fat; purchased from Guangdong Medical Experimental Animal Center) for 2 months and then injected with streptozotocin (STZ, 50 mg/kg/day) for 5 days to induce T2DM; meanwhile, the original high-fat diet was maintained. In a random manner, the animals (*n* = 24) were divided into the following groups: (1) normal group (*n* = 6, all normal control animals were fed normal food); (2) DM model group (*n* = 6); (3) NaB intervention group, DM animals were orally administered NaB (*n* = 6, 0.5 g/kg/day); and (4) positive control group, DM animals were orally administered metformin (et, 0.15 g/kg/day, *n* = 6). All drugs were orally administrated for 16 weeks. At the end of the experiment, mice were killed by cervical dislocation method; the serum, fecal, liver, and gut tissues were collected for further study.

### 2.3. EIA Analysis

Based on the manufacturer’s instructions, the serum or urine biochemical indices (AGEs, IL-6, TNFα, IL-1β, ALT, AST, TBA, T-CHO, TG, LDLC, HDLC) of the mice were determined using an ELISA kit, cell total bile acid (TBA, E003-2-1, Nanjing, Jiangsu, China) was determined using an ELISA kit. Absorbance measurements were performed using a multimode microplate reader (Molecular Devices, San Jose, CA, USA).

### 2.4. Histological Observation

Hematoxylin-eosin (HE) staining was performed on liver and gut paraffin sections that were deparaffinized with xylene and first treated with gradient ethanol, followed by distilled ddH_2_O washing. Deparaffinized tissue sections were stained with hematoxylin and eosin. After ethanol gradient dehydration, xylene transparent was applied, followed by neutral gum sealing.

For periodic acid–Schiff (PAS) staining, fresh tissues were fixed in buffered formalin. Tissue slides were stained with PAS solution followed by Mayer’s hematoxylin.

All slides were finally observed and recorded under an optical microscope (Olympus, Tokyo, Japan).

### 2.5. Western Blot (WB)

Cells or fresh tissues were lysed in 1X RIPA lysis buffer (ab156034, Abcam, Cambridge, MA, USA). Cell or tissue protein was measured with the BCA Protein Assay Kit (P0009, Beyotime Institute of Biotechnology, Shanghai, China) according to the manufacturer’s instructions. Equivalent amounts of protein (35 μg) were separated by different concentrations of SDS–PAGE gels, transferred to polyvinylidene fluoride (PVDF) membranes (IPVH00010, Millipore, Billerica, MA, USA), and then blocked with 5% BSA in Tris-buffered saline containing 0.1% Tween 20 (TBST) for 1 h at room temperature (RT). After being incubated in diluted primary antibodies (GPR43 (bs-13536R), PTPN6 (bs-4158R), GATA4 (bs-1778R), GLUT2 (bs-0351R), Insulin Receptor (bs-0681R), PI3K (bs-10657R), and p-PI3K (bs-6417R), TGR5 (ab72608), β-actin (AF5001), AKT (sc-5298), FXR (sc-25309), p-AKT (sc-293125), CYP7A1 (sc-518007), GSK3α/β (sc-7291), and p-GSK3α/β (sc-81496)) at 4 °C overnight, membranes were further incubated with secondary antibodies (1:10000; Abcam, Cambridge, MA, USA) for 1 h at RT. Finally, the membranes were scanned with an AI800 Imaging System (GE Healthcare, Chicago, IL, USA). The protein band density was normalized to that of the corresponding β-actin and analyzed by ImageJ V1.5.2 software.

### 2.6. Proteomic Analysis

Liver tissues were lysed with lysis buffer (40 mM Tris-HCl, 2 M thiourea, 7 M urea, 4% SDS, pH 8.5) containing 2 mM EDTA and 1 mM PMSF, and then 10 mM DTT was added to the sample. After sonicating on ice, the suspension was centrifuged at 4 °C. Precooled acetone was mixed with the supernatant. A mixture of 8 M urea/100 mM TEAB (pH 8.0) was used to resuspend the protein pellets after centrifugation. Using 10 mM DTT at 56 °C for 30 min, protein samples were reduced at RT for 30 min and then alkylated in the dark for 30 min. The concentration of the total protein was measured. Proteins were digested with trypsin at RT for 12–16 h, a C18 column was used to desalt peptides, and a vacuum concentration (LabTech, Beijing, China) meter was used to dry the desalted peptides. After that, under high pH conditions, samples were fractionated by a high-performance liquid chromatography (HPLC) system Thermo DINOEX Ultimate 3000 BioRS (Thermo Scientific, Waltham, MA, USA). After being dried by vacuum centrifugation (Thermo Scientific Waltham, MA, USA), a Q Exactive plus mass spectrometer (Thermo Scientific, Waltham, MA, USA) was used to analyze all protein samples, along with an UltiMate 3000 RSLC nanosystem (Thermo Scientific, Waltham, MA, USA).

### 2.7. 16S rRNA Sequencing

#### 2.7.1. Sample Preparation

A CTAB/SDS method was used to extract total genomic DNA from samples. The specific primers paired with the barcode were used to amplify 16S rRNA, 18S rRNA, and ITS genes from distinct regions. Purification of PCR products was performed using a Qiagen Gel Extraction Kit (QIAGEN, Hilden, Germany). Following the manufacturer’s instructions, a TruSeq^®^ DNA PCR-Free Sample Preparation Kit (Illumina, CA, USA) was used to generate sequencing libraries. A Qubit 2.0 Fluorometer (Thermo Scientific, Waltham, MA, USA) and an Agilent Bioanalyzer 2100 system (Agilen, Santa Clara, CA, USA) were used to assess library quality. In the final step, paired-end reads of 250 bp were generated using an Illumina NovaSeq platform (Illumina, CA, USA). Supplementary Table S2 shows differentially expressed proteins.

#### 2.7.2. Bioinformatic Analysis

Paired-end reads assembly and quality control: The paired-end reads were split and assigned to samples based on barcodes, and the primer sequence was removed. Then, the reads were merged using FLASH for accurate analysis, and the raw tags were obtained. Quality filtration was performed to obtain high-quality clean tags. Chimera sequences were detected and removed using the UCHIME algorithm, resulting in the acquisition of effective tags.

OTU cluster and Species annotation: The Uparse V7.0.1001 software was used to perform sequence analysis and assign sequences with ≥97% similarity to the same OTUs. Representative sequences for each OTU were selected for further annotation using the Silva Database based on the Mothur algorithm. Phylogenetic relationships among different OTUs were established through multiple sequence alignment using the MUSCLE V3.8 software. The OTUs’ abundance information was normalized, and subsequent alpha and beta diversity analyses were conducted based on this normalized data. Supplementary Figure S3-1 to S3-8 shows 16S rRNA sequencing data.

### 2.8. Cell Culture

At 37 °C in the incubator, HepG_2_ cells were cultured in high-glucose MEM media (Gibco) containing 10% fetal bovine serum (FBS) and 1% penicillin–streptomycin. CaCo_2_ cells were cultured in high-glucose DMEM (Gibco) containing 1% glutamine, 10% fetal bovine serum (FBS), 1% nonessential amino acids, and 1% penicillin–streptomycin at 37 °C in an incubator.

### 2.9. CCK8 Assay

A CCK8 Kit (Beyotime, Shanghai, China) was used for the cell viability assay. At a density of 5 × 10^3^ cells per well, the cells were seeded into 96-well plates. The plates were incubated at RT for 1.5 h. A plate reader (Molecular Devices, USA) was used to measure 490 nm absorbance values.

### 2.10. SiRNA Transfections

In 6-well plates, HepG_2_ cells (3.5 × 10^5^ cells/well) were seeded for 24 h before transfection and replaced with fresh medium without serum or antibiotics after 24 h. HepG_2_ cells were transfected with 25 pmol/well SiGPR43 or negative control (Santa Cruz) with Lipofectamine™ RNAiMAX Transfection Reagent (Invitrogen, Carlsbad, CA, USA) according to the manufacturer’s instructions. The medium was replaced after 6 h of transfection, and after 24 h of culture medium incubation, the cells were collected.

### 2.11. PCR Array

A quantitative PCR array analysis of mitochondrial energy metabolism was performed using a QIAGEN RT^2^ Profiler^TM^ PCR Array Mouse Mitochondrial Energy Metabolism (QIAGEN, Hilden, Germany). Total RNA from liver tissue was extracted by TRIzol (Invitrogen, Waltham, MA, USA); purified RNA was converted into cDNA using ReverTra Ace^®^ qPCR Master Mix (FSQ-201, Toyobo, Osaka, Japan). According to the manufacturer’s instructions, PCR arrays were analyzed using Life Technologies’ ViiATM 7 high-productivity real-time quantitative PCR kit (Gaithersburg, MD, USA). An analysis of gene expression fold changes was performed using the 2^−ΔΔCt^ method.

### 2.12. Q-PCR

We extracted total RNA from HepG_2_ cells using TRIzol reagent and measured its concentration and quality using a NanoDrop 2000 (Wilmington, DE, USA). A reverse transcriptase kit (FSQ 101) from TOYOBO was used for reverse transcription. On a ViiATM 7 Real-Time Detection System (Life Technologies, Gaithersburg, MD, USA) using SYBR Green qPCR Master Mix (QIAGEN, Hilden, Germany), quantitative real-time PCR was performed. By using the 2^−ΔΔCt^ method, the relative expression of genes was compared to that of β-actin. Supplementary Table S1 shows primer sequences.

### 2.13. Immunofluorescence

Cells were seeded into a 6-well plate, treated with 4% paraformaldehyde, and further perforated with 0.5% Triton X-100. After blocking with 5% BSA, a primary antibody was incubated with cells overnight at 4 °C, followed by a secondary antibody incubation at 37 °C for one hour in the dark. DAPI was applied for nuclear staining. Cells were finally observed and recorded under a confocal laser scanning microscope (Leica TCS SP8, Wetzlar, Germany).

### 2.14. Live-Cell Imaging of HepG2 Cells

HepG_2_ cells were treated with mitochondria MitoTracker™ Deep Red FM and imaged using a live-cell imaging system (Leica, Germany). Multiple positions within the same time course were imaged using point visits at 37 °C and 5% CO_2_.

### 2.15. Transepithelial Electrical Resistance (TEER) Measurement of CaCo_2_ Monolayers

As a measure of the integrity of CaCo_2_ cell–cell junctions, electrical resistance values were used. Cells grown on Costar Transwell polyester membrane inserts with a pore size of 5 μm were treated with high concentrations of insulin and lipopolysaccharide (LPS). TEER readings were recorded before and after 24 h of drug treatment. A Millicell ERS-2 Volt-Ohm meter (EMD Millipore, Merck Millipore, Burlington, MA, USA) was placed into both the basal and apical chambers of the transwells, there was a current passing through the monolayers, and data on the resistance in Ω.cm^2^ were reported. In addition to the membrane surface area (0.33 cm^2^), a correction was made for the membrane’s electrical resistance (blank transwell).

### 2.16. Statistical Analysis

Analysis of variance was performed using one-way analysis of variance in GraphPad Prism 7.0 to compare data among groups unless otherwise indicated. Data are expressed as the mean ± SEM. Data were analyzed with ImageJ V1.5.2 software. A *p* value of 0.05 indicates a significant difference between the groups.

## 3. Results

### 3.1. NaB Ameliorated Metabolic Dysfunction in Diabetic Mice

**Figure 1:**
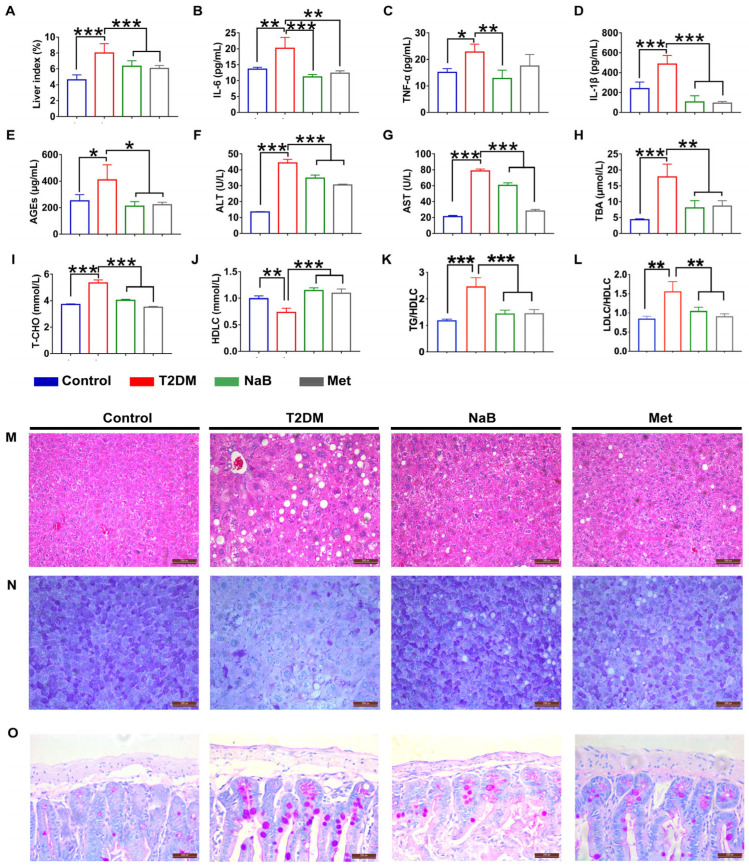
NaB ameliorated liver and intestine injury in T2DM mice. (**A**) The liver index of mice in each group. The levels of (**B**) IL-6, (**C**) TNF-α, (**D**) IL-1β, (**E**) AGEs, (**F**) ALT, (**G**) AST, (**H**) TBA, (**I**) T-CHO, (**J**) HDLC, (**K**) TG/HDLC, and (**L**) LDLC/HDL-C were determined by ELISA. * *p* < 0.05, ** *p* < 0.01, *** *p* < 0.001, *n* = 6. (**M**) Representative images of hematoxylin and eosin (HE)-stained liver tissues; (**N**) PAS staining of liver; (**O**) HE staining of small intestine. Magnification: 400×.

Metabolic dysfunction is a common phenomenon that seriously promotes the development of diabetes. In the present study, the levels of liver index (Figure 1A), advanced glycation end products (AGEs, Figure 1E), total cholesterol (T-CHO, Figure 1I), the radio of triglyceride to high-density lipoprotein-cholesterol (TG/HDLC, Figure 1K), the radio of low-density lipoprotein-cholesterol to high-density lipoprotein-cholesterol (LDLC/HDLC, Figure 1L), and blood aspartate aminotransferase (ALT and AST) (Figure 1F,G) in diabetic mice were strikingly elevated (*p* < 0.01, vs. control), and the level of high-density lipoprotein-cholesterol (HDLC, Figure 1J) in diabetic mice was significantly reduced (*p* < 0.01, vs. control), while NaB administration ameliorated metabolic dysfunction of the above parameters. Interestingly, we also proved that NaB supplementation decreased blood total bile acid (TBA) in diabetic mice (Figure 1H). Low-grade inflammation is also recognized as a complication of diabetes. In this study, we found that the levels of serum interleukin 6 (IL-6, Figure 1B), tumor necrosis factor α (TNF-α, Figure 1C), and interleukin 1β (IL-1β, Figure 1D) were dramatically increased compared with the control, while NaB administration decreased their levels. These data suggested that NaB could ameliorate both diabetic inflammation and metabolic dysfunction.

### 3.2. Histological Observation of Liver and Intestine Tissues in Diabetic Mice

To observe changes in the intestine–liver axis, histological structure was studied. As shown in Figure 1M,N, there was no infiltration of inflammatory cells or necrosis of hepatocytes in the normal group, and liver lobules and hepatocytes were clearly arranged. However, in the model group, several hepatocytes were necrotic, and the lobular structure of the liver was destroyed. In comparison with the model group, the NaB group showed a lesser degree of hepatocyte necrosis.

The intestine plays a pivotal role in metabolic diseases. As shown in Figure 1O, in the model group, the intestinal permeability was markedly increased compared with that in the normal group, while NaB treatment dramatically enhanced the integrity of the intestine compared with that in the model animals (Figure 1O).

### 3.3. NaB Can Improve the Abundance of Intestinal Flora in Diabetic Mice

**Figure 2:**
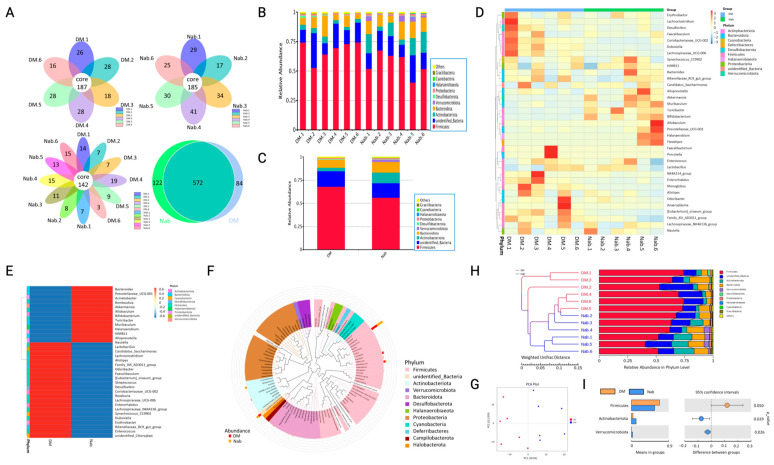
NaB improved intestinal flora diversity in mice. (**A**) Venn diagram and petal diagram based on OTU, (**B**,**C**) histogram of relative abundance of species at the phylum level, (**D**,**E**) species abundance cluster map, (**F**) genus-level species tree, (**G**) principal component analysis, (**H**) UPGMA clustering tree based on weighted UniFrac distance, (**I**) *t* test analysis of species differences between DM and NaB groups. *n* = 6.

It is believed that the cometabolism between the organism and intestinal flora plays a key role in the development of diabetes. It would be interesting to know whether NaB supplementation, as a metabolic product of microbiota, has any influence on gut microbiota composition. As shown in Figure 2, diabetic animals had a reduced diversity of microbiota since their microbiota composition was different between groups. By analyzing specific microbiota compositions, we observed that *Firmicutes* and *Bacteroidetes* were the most intestinal flora in mice. In addition, we observed a significant increase in *Actinobacteria* in diabetic mice after NaB treatment. (Figure 2B,C); in diabetic mice, the abundance of *Bacteroidetes* and *Actinobacteriota* was reduced, while *Firmicutes* was significantly increased, and NaB administration significantly increased *Bacteroidetes* and *Actinobacteriota* and reduced Firmicutes (Figure 2H). The representative sequences of the top 100 genera were aligned using multiple sequence alignment to study phylogenetic relationships among species at the genus level (Figure 2F). As shown in Figure 2F, *Lactobacillus*, *Dubosiella*, *Facecalibaculum*, and *Monoglobus* of the *Firmicutes* were detected in the DM group, while *Lactobacillus*, *Turicibacter*, *Allobaculum*, *Facecalibaculum*, and *Lachnospiraceae_NK4A136_group* of the *Firmicutes* were detected in the NaB group. Compared with the DM group, the abundance of *Lactobacillus* did not change in the NAB group; the abundance of *Dubosiella*, *Facecalibaculum*, and *Monoglobus* decreased; and the abundance of *Allobaculum*, *Turicibacter*, and *Lachnospiraceae_NK4A136_group* increased. *Bifidobacterium*, *Enterorhabdus*, and *Coriobacteriaceae_UCG-002* of the *Actinobacteriota* were detected in the DM group, while *Bifidobacterium* was detected in the NaB group. Compared with the DM group, the abundance of *Bifidobacterium* significantly increased in the NAB group. *Alistipes* and *Bacteroides* of the *Bacteroidetes* were detected in the DM group, while *Alistipes*, *Alloprevotella*, and *Bacteroides* was detected in the NaB group. Compared with the DM group, the abundance of *Alloprevotella* significantly increased in the NAB group, the abundance of *Alistipes* and *Bacteroides* were significantly reduced in the NAB group. Principal coordinate analysis (PCoA) was carried out, and significance was found between the model and NaB administration groups (Figure 2G). From the species abundance at different levels, differential species were obtained by conventional t tests. There was a significant *p* value for the abundance of *Firmicutes* in both the model and NaB groups, and the average abundance of *Firmicutes* in the NaB group was 56.1% and in the model group was 68.1% (Figure 2I).

### 3.4. NaB Ameliorated the High Insulin + LPS-Induced Increase in Transendothelial Albumin Passage in CaCo_2_ Cells

**Figure 3:**
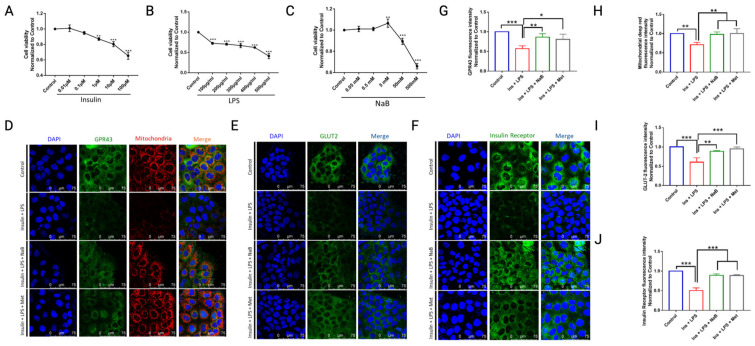
NaB ameliorated high glucose- and LPS-induced damage in CaCo_2_ cells. (**A**) Insulin, (**B**) LPS, and (**C**) sodium butyrate (NaB) concentration-dependently inhibited CaCo_2_ cell viability, * *p* < 0.05, ** *p* < 0.01, *** *p* < 0.001, vs. control, *n* = 3. Immunofluorescence assays for (**D**) GPR43, (**E**) GLUT2, and (**F**) insulin receptor (IR) under a laser scanning confocal microscope and (**G**–**J**) relative fluorescence intensity were determined. * *p* < 0.05, ** *p* < 0.01, *** *p* < 0.001, vs. Ins + LPS, *n* = 3.

To explore the effects and mechanism of NaB in gut–liver axis-modulated bile acid metabolism, we used CaCo_2_ cells to represent the gut in vitro. As CaCo_2_ cells possess strong regenerative ability, our first step was to exclude the influence of cell amount on glycogen determination within CaCo_2_ cells. In Figure 3A–C, we can see that cell viability was prevented by LPS, insulin or NaB in a concentration-dependent manner after incubation for 24 h. Converging reports [17] and our present observation (Figure 3A–C), we finally applied 1 μmol/mL insulin plus 100 μg/mL LPS (Ins + LPS) and 5 mM NaB to stimulate CaCo_2_ cells in the following research unless otherwise noted.

In the present study, under high insulin and LPS settings, we observed that the expression of glucose transporter 2 (GLUT2) and insulin receptor was substantially decreased, while NaB inhibited this decrease (Figure 3D–J). To investigate the underlying mechanism, the protein expression of the receptor for short-chain fatty acids (SCFAs), GPR43, was determined. We found that Ins + LPS decreased the expression of GPR43 in CaCo_2_ cells, and NaB reversed this reduction. This finding obviously suggests that NaB may mediate glycogenesis via GPR43. Mitochondria have been well recognized in mediating metabolism during the development of diabetes. In the study, we also found that both GPR43 expression and mitochondria with membrane potential were decreased under high insulin and high LPS stimulation (Figure 3D,G,H), and NaB treatment ameliorated mitochondrial damage via GPR43.

**Figure 4:**
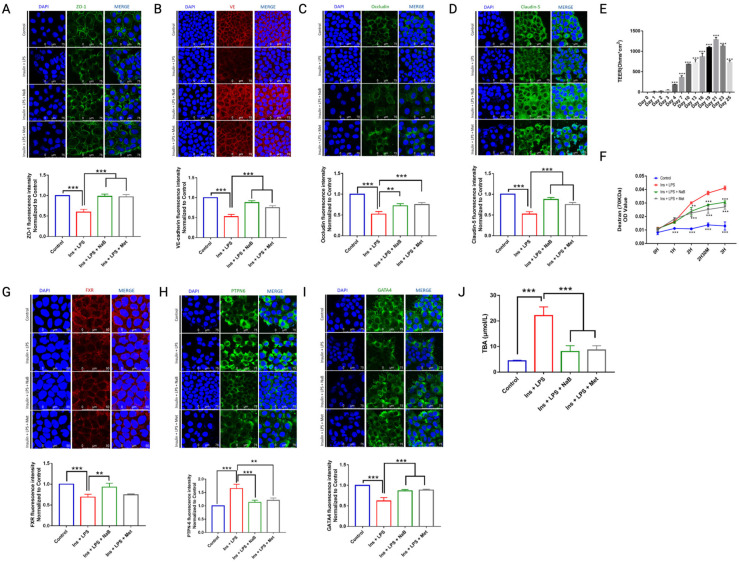
Monocellular barrier function analysis in CaCo_2_ cells. Immunofluorescence assay for (**A**) ZO-1, (**B**) VE, (**C**) Occludin, (**D**) Claudin-5, (**G**) FXR, (**H**) PTPN6, and (**I**) GATA4 under a laser scanning confocal microscope, and fluorescence intensity was determined relative to each other. (**E**) The TEER of CaCo_2_ cells was increased from Day 0 to Day 21; (**F**) Insulin + LPS increased transendothelial albumin passage across the monocellular barrier; ** *p* < 0.01, *** *p* < 0.001, vs. control (Day 0 or 0H). Intracellular content of TBA in (**J**) CaCo_2_ was determined by kit; *** *p* < 0.001, *n* = 3.

To estimate the influence of treatment with NaB on the membrane integrity of differentiated CaCo_2_ cell monolayers, the TEER (trans-epithelial electrical resistance), which affects the permeability of molecules (both noncharged or charged) through tight junctions, which portrays the paracellular flux, and the presence of a complex of tight junction proteins, were assayed. To investigate the mechanism underlying the inhibition of paracellular transport across NaB-treated CaCo_2_ cell monolayers, the expression of tight junction (TJ)-related proteins was researched by immunofluorescence. As shown in Figure 4A–D, NaB treatment enhanced TJ proteins, including occludin, VE, claudin-1, and ZO-1, compared with high insulin and LPS stimulation of CaCo_2_ cells. Since diabetes has been demonstrated to be accompanied by increased permeability of the intestinal barrier, we examined whether NaB was able to preserve the permeability of cultured CaCo_2_ cell monolayers. We found that the TEER value of CaCo_2_ cells gradually increased until the 21st day (Figure 4E), so the mono-cellular model was established on the 21st day of cell culture. The permeability was assessed by measuring the passage of 70 kDa dextran across the monolayer, as presented in Figure 4F. Stimulation with high insulin + LPS increased transendothelial albumin passage across CaCo_2_ cells, while this was ameliorated in the presence of NaB.

As most bile acids were reabsorbed within the intestinal lumen, we further investigated changes in related proteins within CaCo_2_ cells. We found that NaB significantly upregulated FXR (Figure 4G) and GATA4 (Figure 4I) but reduced PTPN6 (Figure 4H) levels compared with cells treated with high insulin + LPS. Moreover, total bile acids (TBA) within CaCo_2_ cells were reduced by NaB (Figure 4J).

### 3.5. NaB Regulated Bile Acid Metabolism-Related Receptors in the Liver

**Figure 5:**
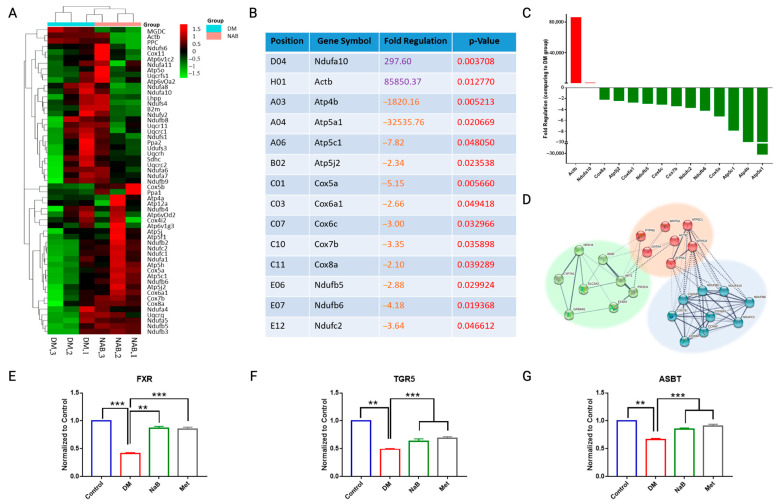
NaB enhanced mitochondrial energy metabolism. (**A**–**C**) The RT^2^ Profiler PCR Array was used to analyze changes in mitochondrial energy metabolism genes; (**A**) Heatmap plot for fold changes in genes between the DM and NaB groups; (**B**,**C**) fold changes in gene expression for 14 representative genes after administration of NaB. The purple color in (**B**) represents a positive Fold regulation value. The orange color in (**B**) represents a negative Fold regulation value. The red color in Figure B represents the *p*-value. (**D**) Protein–protein interactions predicted from the STRING database among mitochondrial energy metabolism genes and NaB-activating genes. (**E**) FXR and (**F**) TGR5 expression in liver tissues and (**G**) ASBT expression in intestinal tissue were analyzed by Q-PCR. ** *p* < 0.01, *** *p* < 0.001, vs. DM, *n* = 3.

In our previous study, we proved that glycogen metabolism was promoted by NaB [17], enhanced mitochondrial function [23], and reduced the blood glucose level in db/db mice [21]. To further screen related genes within this modulation, a PCR array kit was used to determine changes in gene expression related to mitochondrial energy metabolism. As shown in Figure 5A–C, 12 genes were significantly downregulated, and 2 genes were substantially upregulated in the livers of NaB-treated DM mice.

A protein–protein interaction (PPI) analysis of genes in the STRING database was conducted to predict possible mechanisms. As depicted in Figure 5D, bile acid metabolism-related receptors play a crucial role in modulating the changes in mitochondrial genes, and receptors for SCFAs may alter the balance of bile acid metabolism. In Figure 5D, we found that CYP7A1 and GATA4 were involved in protein interactions. These genes are key receptors involved in bile acid metabolism. The main function of the CYP7A1 gene is to catalyze the conversion of cholesterol into bile [24]. The loss of GATA4 in the intestine will lead to bile acid reabsorption [25]. By Q-PCR, we verified that the gene expression of FXR and TGR5 was upregulated in the liver tissue of NaB-administered mice and that ASBT was upregulated in the intestinal tissue of NaB-administered mice. To further demonstrate the above prediction, omics were carried out in the following study.

### 3.6. Proteomic Study of the Liver in Mice

**Figure 6:**
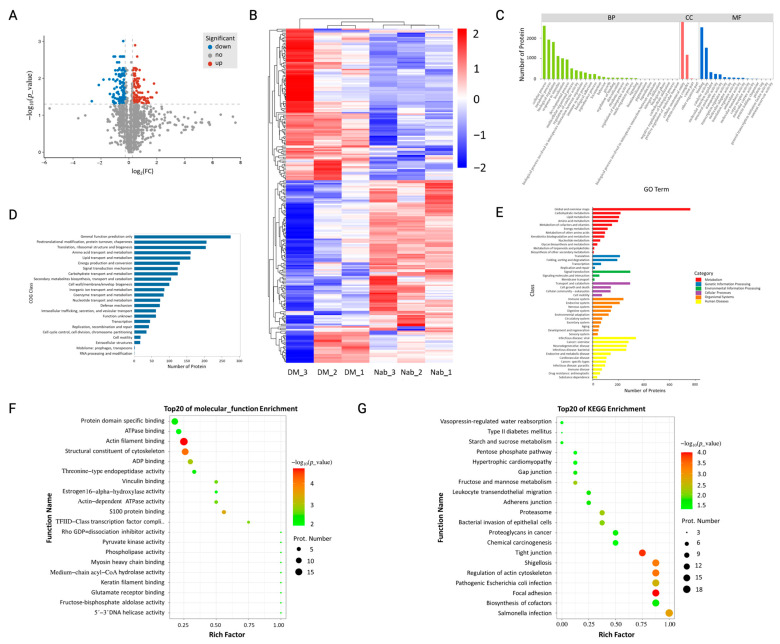
Proteomics results in mouse livers. (**A**) Protein FC and FDR volcano plot distribution, (**B**) hierarchical clustering heatmap of differentially expressed proteins, (**C**) Gene Ontology annotation, (**D**) Cluster of Orthologous Groups of proteins Annotation, (**E**) KEGG pathway annotation result statistics, (**F**) isoprotein GO enrichment molecular function analysis bubble chart, (**G**) pathway metabolic pathway enrichment. *n* = 3.

To investigate differential protein expression between the DM model and NaB administration groups, proteomic analysis of the liver was performed. A total of 2926 proteins were identified through quantitative proteomic studies combining LFQ and LC–MS/MS techniques. As shown in Figure 6A, compared with the diabetic group, we found that 108 proteins were upregulated, while 132 were downregulated. By hierarchical clustering (Figure 6B) and Gene Ontology (GO) analysis (Figure 6C), a significant difference in protein expression was found between the two groups. To explore orthologous classification of differentially expressed proteins and their coordination, cluster of orthologous groups of proteins (COG) (Figure 6D) and pathway-based analysis (Figure 6E) were carried out. We found metabolic dysfunction and energy production differences between the two groups.

To further understand the functions of the proteins, differential protein function enrichment analysis was conducted. Molecular function enrichment analysis (Figure 6F) showed that actin filament binding, ADP binding, actin-dependent ATPase activity, protein domain-specific binding, threonine-type endopeptidase activity, glutamate receptor binding, fructose-bisphosphate aldolase activity, phospholipase activity, pyruvate kinase activity, medium-chain acyl-CoA hydrolase activity, estrogen 16-alpha-hydroxylase activity, and ATPase binding were the most important molecular functions. Differential protein pathway enrichment analysis (Figure 6G) indicated that tight junctions, fructose and mannose metabolism, biosynthesis of cofactors, gap junctions, type II diabetes mellitus, starch and sucrose metabolism, and the pentose phosphate pathway were the most important pathways.

### 3.7. NaB Ameliorated High Glucose- and LPS-Induced Damage in HepG2 Cells via GPR43

**Figure 7:**
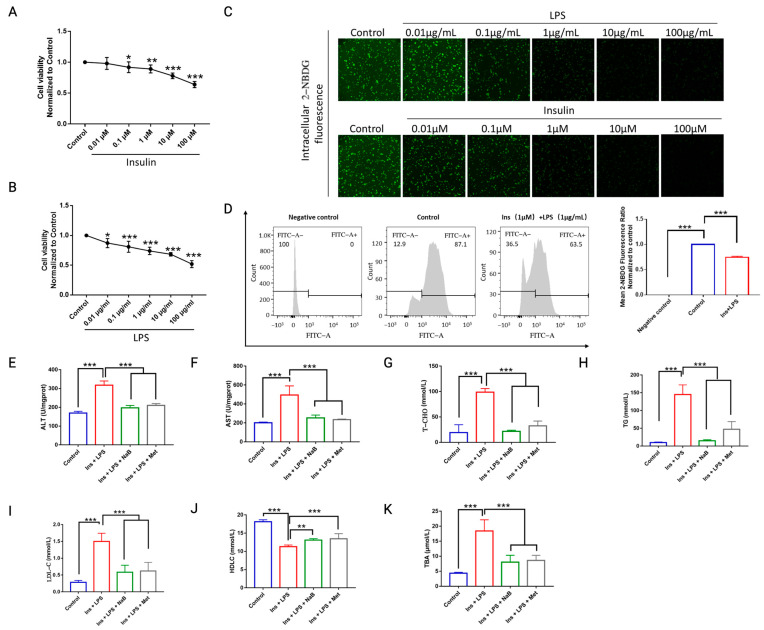
NaB ameliorated high glucose- and insulin-induced damage in HepG_2_ cells. (**A**) Insulin and (**B**) LPS concentration-dependently reduced HepG_2_ cell viability. * *p* < 0.05, ** *p* < 0.01, *** *p* < 0.001, vs. control. (**C**) Intracellular density of 2-NBDG fluorescence was observed among groups. (**D**) Flow cytometry of HepG_2_ cell glucose uptake among groups, *** *p* < 0.001. Metabolic function analysis of cholesterol and lipoproteins in HepG_2_ cells, the levels of (**E**) ALT, (**F**) AST, (**G**) T-CHO, (**H**) TG, (**I**) LDL-C, (**J**) HDL-C, and intracellular content of TBA within (**K**) HepG_2_ was determined by kit, * *p* < 0.05, ** *p* < 0.01, *** *p* < 0.001, *n* = 3.

To explore the effects and underlying mechanism of NaB in gut–liver axis-modulated bile acid metabolism, we used HepG2 cells in the following study. To eliminate the influence of cell amount on glycogen determination within HepG2 cells, we first incubated the cells with insulin or LPS and found that a dose-dependent inhibition of cell viability was observed after 24 h incubation with insulin or LPS (Figure 7A,B). We stimulated the cells with high insulin combined with LPS, and we further verified the functions of the cells on glucose uptake by detecting 2-NBDG using immunofluorescence microscopy (Figure 7C) and flow cytometry (Figure 7D). Based on previous reports [17] and our present observations (Figure 7A–D), we finally applied 1 μmol/mL insulin and 1 μg/mL LPS (Ins + LPS) to stimulate HepG2 cells, and 0.5 mM NaB was administered to observe its effect, unless otherwise stated.

To further verify whether NaB could moderate lipid metabolism under diabetic conditions, lipid levels were assayed in HepG2 cells. We found that NaB significantly reduced the levels of total cholesterol (T-CHO, Figure 7G), total triglycerides (TG, Figure 7H), and low-density lipoprotein cholesterol (LDL-C, Figure 7I) compared with the model group (Ins + LPS) and increased high-density lipoprotein cholesterol (HDL-C, Figure 7J). We further found that NaB reduced the levels of alanine aminotransferase (ALT, Figure 7E) and aspartate aminotransferase (AST, Figure 7F) induced by Ins + LPS, thus exerting protective effects against cell damage. In line with the in vivo findings (Figure 1H), NaB reduced TBA production in HepG2 cells (Figure 7K).

**Figure 8:**
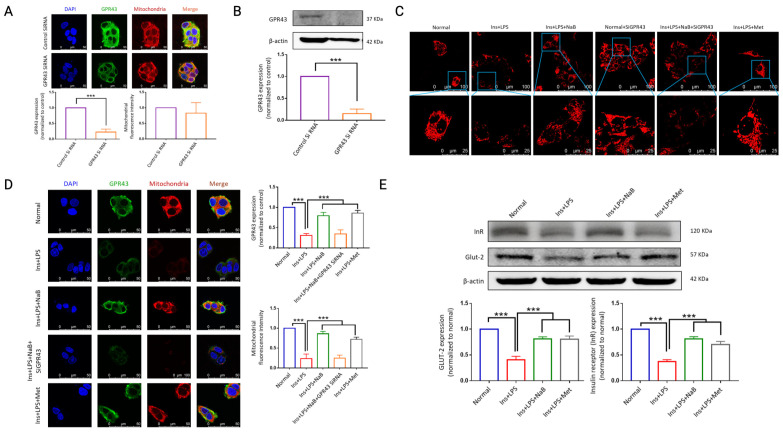
NaB improves mitochondrial function and energy metabolism in HepG_2_ cells Via GPR43. (**A**) An immunofluorescence assay was performed on siRNA-GPR43 and mitochondria under a laser scanning confocal microscope, and the relative fluorescence intensities were measured. (**B**) Western blot analysis for GPR43 and β-actin, *** *p* < 0.001. (**C**) MitoTracker™ Deep Red FM was used to stain the mitochondria of HepG_2_ cells for confocal imaging. (**D**) Immunofluorescence microscopy and confocal microscopy were used to analyze GPR43 and mitochondrial localization and expression. (**E**) Western blot analysis of GLUT-2 and insulin receptor (InR) was performed. Immunofluorescence assay for GLUT-2. *** *p* <0.001, *n* = 3. Supplementary Figure S1 show the native image of Western blot of Figure 8.

Mitochondria have been well recognized in mediating metabolism during the development of diabetes, and GPR43 is a recognized receptor for SCFAs. To verify the role of GPR43 in NaB-mediated metabolism, its expression was first silenced by GPR43 siRNA and double-checked by immunofluorescence (Figure 8A) and WB (Figure 8B) in HepG2 cells. We found that silencing GPR43 had no direct effect on mitochondria (Figure 8A). Immunofluorescence observation showed that both GPR43 expression and mitochondria with membrane potential were increased by NaB (Figure 8C,D), while silencing GPR43 significantly inhibited the effects of NaB on mitochondria, as detected by live cell imaging (Figure 8C). In mediating hepatocyte glucose uptake, glucose transporters as well as receptor for insulin (InR) play important roles. In our present research, we found that the expression of glucose transporter 2 (GLUT-2) and InR was significantly reduced under high insulin and LPS settings, suggesting significant glucose uptake dysfunction, while NaB administration significantly inhibited this decrease (Figure 8E). The above findings clearly suggest that NaB mediates protect mitochondria and hepatocyte glycogenesis Via GPR43.

### 3.8. NaB Ameliorated Lipid Metabolism Dysfunction in HepG2 Cells

**Figure 9:**
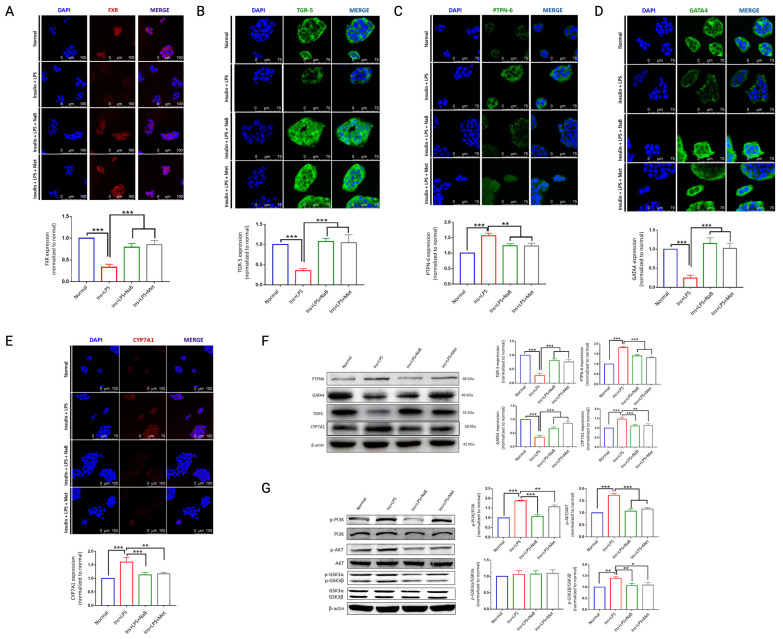
Effect of NaB on bile acid metabolism-related proteins in HepG_2_ cells. Sodium butyrate (NaB) increased FXR, TGR5, PTPN6, GATA4, and CYP7A1 expression. Immunofluorescence assay for (**A**) FXR, (**B**) TGR5, (**C**) PTPN6, (**D**) GATA4, and (**E**) CYP7A1 under a laser scanning confocal microscope, and fluorescence intensity was determined relative to each other. (**F**) The above proteins were analyzed using Western blotting. The PI3K/AKT signaling pathway participated in the function of NaB. (**G**) WB detection of PI3K/AKT/GSK3 signaling proteins, including the ratio of p-PI3K to total PI3K, ratio of p-AKT to total AKT, and ratio of p-GSK3α to total GSK3α; (**E**) ratio of p-GSK3β to total GSK3β. * *p* < 0.05, ** *p* < 0.01, *** *p* < 0.001, vs. Ins + LPS, *n* = 3. Supplementary Figure S2 shows the native image of the Western blot of Figure 9.

In addition to synthesizing and secreting bile acid (BA), hepatocytes also play a critical role in fat metabolism and glucose metabolism. In this study, we found that NaB could significantly reduce TBA induced by high Ins + LPS (Figure 1H and Figure 7K). Takeda G-protein coupled receptor 5 (TGR5) and farnesoid X receptor (FXR) are known BA-sensitive receptors in both intestine and liver tissues. In the in vivo study, we found that the expression of FXR, TGR5 and ASBT was significantly reduced in diabetic animals, and either NaB or Met administration upregulated their expression (Figure 5E–G); this was further verified in HepG2 cells (Figure 9A,B) by immunofluorescence. We also found that NaB upregulated GATA-4 (Figure 9D) while downregulating PTPN-6 (Figure 9C) and CYP7A1 (Figure 9E) in Ins + LPS-induced hepatocytes by both immunofluorescence and WB (Figure 9F).

To further explore the underlying mechanism, classic intracellular signaling pathways were studied. As shown in Figure 3G, we found that Ins + LPS stimulation significantly increased the phosphorylation of PI3K-AKT-GSK3β proteins within hepatocytes, and NaB administration blocked their activation, suggesting that PI3K/Akt/GSK3β may participate in NaB-modulated metabolism in cells.

## 4. Discussion

A complex disease characterized by excessive liver glucose production, T2DM occurs when the liver produces too much glucose, insulin resistance, and reduced glucose processing capacity in muscles and adipocytes. Metabolic syndrome and T2DM are directly related to an increase in the number of obese populations. The liver plays a pivotal role in modulating metabolism. Scholars have drawn attention to the role of bile acid in disease progression, as we understand more about the mechanisms of type 2 diabetes. Bile acids are produced by liver cells and are crucial for lipid digestion and absorption. Our previous research [26] indicated their role in controlling the progression of diabetes and modulating glucose metabolism. Based on the findings of this study, we further demonstrated that a metabolite from the intestinal flora, butyrate, modulated the progression of T2DM by modulating the metabolism of bile acids, and its effects on the gut–liver axis were the underlying mechanism.

Recently, studies have revealed that the intestinal flora is related to a variety of diseases, including intestinal tumors, obesity [27], and diabetes [28], as the study of the intestinal flora becomes increasingly intensive. An imbalance in the gut microflora is apparent in diabetes [29] and obese patients. In our study, we found that *Firmicutes* was the most dominant bacterial phylum in the intestinal flora of diabetic mice. The *Lactobacillus*, *Dubosiella*, *Facecalibaculum* and *Monoglobus* of the *Firmicutes* were found in the DM group. Among these genera, *Dubosiella*, *Facecalibaculum* and *Monoglobus* can all produce butyrate. The *Lactobacillus*, *Facecalibaculum*, *Turicibacter*, *Allobaculum* and *Lachnospiraceae_NK4A136_group* of the *Firmicutes* were detected in the NaB group. Among these genera, *Turicibacter*, *Allobaculum*, *Facecalibaculum* and *Lachnospiraceae_NK4A136* can all produce butyrate. This indicates that both the DM and NaB groups in our study obtained additional sources of butyrate produced by the Firmicutes bacteria, but their butyrate came from different genera. Wang et al. used a genome-wide association study (MGWAS) to show that patients with type 2 diabetes have a moderate intestinal dysbiosis, with a decrease in the abundance of some universal butyric acid-producing bacteria and an increase in the abundance of various opportunistic pathogens, they also found in T2DM population that the abundance of butyrate-producing bacteria was reduced [30]. In our previous study, we found oral supplementation with sodium butyrate in db/db mice can significantly enrich diversity of the intestinal bacteria and ameliorate dysfunction of metabolisms [17].

According to the current study, the abundance of *Bacteroidetes* and *Actinobacteriota* was downregulated, while *Firmicutes* was upregulated in diabetic mice, and NaB administration significantly upregulated *Bacteroidetes* and *Actinobacteriota* and downregulated *Firmicutes*. In our study, we found that *Firmicutes*, *Bacteroidetes*, and *Actinobacteriota* were the predominant intestinal microbiota in diabetic mice. While *Firmicutes* and *Bacteroidetes* are typically the dominant group in the mouse gut, *Actinobacteriota* also made up a significant portion of the microbiota in our study, potentially related to the diabetic mouse model we studied. Interestingly, we observed a significant increase in the abundance of *Actinobacteriota* after NaB treatment. A systematic review by Ethan Slouha et al., analyzing multiple studies, found that *Actinobacteriota* was significantly more abundant in healthy subjects compared to those with diabetes [31], consistent with our findings. This suggests that NaB may treat diabetic mice by increasing *Actinobacteriota* abundance. To date, no studies have investigated NaB’s role in improving diabetic mice through improvements in *Actinobacteriota* abundance, and we will continue to explore this topic in the future. A study found patients with type 2 diabetes have been found to have significant associations with specific gut microbes, bacterial genes and metabolic pathways when analyzed using metagenome-wide association studies [32]. It is important to recognize that the gut microbiota is a dynamic entity influenced by lifestyle, antibiotics, diet and genetic background. Host health is correlated with gut microbiota diversity and stability. Diabetic patients showed higher levels of Lactobacillus spp. compared to nondiabetic people. Here, we found that NaB further enriched the composition of bacteria that produce short-chain fatty acids (SCFAs).

In fact, SCFAs, mainly butyrate, propionate, and acetate, are a class of metabolic products from the intestinal flora. SCFAs in the human intestine are an organic acid produced by the fermentation of dietary fiber by intestinal flora. SCFAs are primarily produced in the human colon, with butyrate accounting for 15%. Because the PH of the proximal colon is lower, it promotes the formation of butyrate [33]. Current studies show that SCFAs possess a variety of downstream regulatory mechanisms through which they exert their functions, including G-protein-coupled receptors (GPRs), histone deacetylases (HDACs), and metabolic integration [34]. GPR43, GPR41, GPR109A and Olfr78 have been recognized as receptors for SCFAs. Our study found that GPR43 is significantly distributed in liver and gut tissue; its expression decreased significantly in IR + LPS-stimulated HepG2/CaCo_2_ cells and diabetic mice, and NaB supplementation attenuated this reduction. As NaB was orally administered in the current study, we further investigated its influence within the intestine, specifically, its effect on the gut barrier. Tight junctions consist of some transmembrane proteins, such as claudins and occludin that undergo paracellular diffusion and mediate adhesion as well as barrier formation, which localize to the apical end of the lateral surface of adjacent epithelial cells [35]. As part of the intestinal wall barrier, they play a very important role. It was found that changing these junctions can increase intestinal mucosal permeability [36]. In our study, we found that NaB administration significantly promoted the protein expression of these tight junctions.

The administration of NaB orally [37] or intraperitoneally [23] to diabetic mice has been shown to have protective effects in previous studies. Modulation of energy metabolism might play a crucial role in how it affects liver function, we hypothesized. There is a significant link between diabetes and reduced mitochondrial function. T2DM patients often suffer from liver dysfunction associated with non-alcoholic fatty liver disease. In this condition, diabetic patients’ liver energy substrate metabolism and mitochondrial function are impaired, and their liver cell mitochondria often exhibit decreased mitochondrial maximal respiration and increased mitochondrial uncoupling [38]. One study found decreased ATP content and turnover in the livers of T2DM patients, further demonstrating that mitochondrial damage can affect normal liver function [39]. Our study found that NaB can improve the Ins + LPS-induced decrease in HepG2 mitochondrial membrane potential, suggesting that NaB can protect normal mitochondrial function in liver cells. Sodium butyrate supplementation could ameliorate diabetic-endotoxemia in db/db mice Via restoring composition of gut microbiota and preserving gut epithelial barrier integrity [21]. Reports have suggested that when the human body suffers from diabetes, hyperglycemia can significantly disturb the balance of mitochondrial fusion and fission, and this disturbance is closely related to a variety of chronic complications of diabetes, but the specific mechanism is still unclear [40]. In our study, NaB significantly improved mitochondrial function Via GPR43.

Insulin receptor plays a very important role in insulin signaling and thereafter energy metabolism, while insulin resistance is exhibited with no response to insulin stimuli [41]. The liver is a pivotal organ that modulates the metabolism of both glucose and lipids. However, its function is always discounted under diabetic settings, and insulin resistance is one of its characteristics. Glycogen and TG levels in liver tissue are strongly correlated with the severity of insulin resistance [42]. GLUT2 is the main glucose transporter on the membrane of hepatocytes and is also indispensable for the absorption of glucose in the intestine. Inactivation of GLUT2 leads to the inhibition of glucose uptake and glucose-stimulated insulin secretion [43]. In the present study, we can see that under the condition of high insulin and LPS-induced diabetic endotoxemia, the protein expression of GLUT2 was suppressed, while NaB promoted the expression of GLUT2. By histological observation, we further demonstrated that steatosis and lipid accumulation within the liver were ameliorated by NaB administration. This evidence obviously suggested that NaB reduced insulin resistance in the liver under diabetic conditions.

Postprandial absorption of nutrients is facilitated by bile acids. By activating the G protein-coupled receptor TGR5 and the farnesoid X receptor (FXR), bile acids play an important role in regulating energy metabolism, glucose, and lipids. Clinical studies have shown that metabolic diseases, including diabetes, disrupt bile acid homeostasis, and patients with obesity and/or diabetes have altered serum bile acids [44]. At present, serum bile acid levels have become a biomarker for the diagnosis of diabetes, obesity, and liver diseases [45]. With the understanding of the pivotal role of bile acids in diabetes, the underlying mechanism has been increasingly recognized. In recent years, TGR5 signaling, which is activated by bile acid, has gained much attention as a key mechanism for regulating glucose metabolism and energy expenditure, and their interaction was found to protect against hyperglycemia, diabetes, and obesity; in addition, it was also found that signaling by bile acids can occur through TGR5 in the intestine [46]. In this sense, the bile acid-TGR5 interaction plays an important role in gut–liver axis-modulated diabetes development. An in vivo study demonstrated that under diabetic conditions, CYP7A1 expression was upregulated [45]. In fact, bile acids can be transported into a cell by the bile acid transporter ASBT; within hepatocytes, bile acids can be further metabolized as the endogenous substrate for FXR. Under diabetic conditions, we found a significant reduction in ASBT, TGR5, and FXR expression in the present study, and administration of NaB improved the metabolism of bile acids. It is well known that the elevation of AST, ALT, TC, TG, and LDL-C levels as well as the decrease in HDL-C levels are common manifestations of hepatocyte injury [47,48,49]. By administering NaB and Met to T2DM-induced mice, blood glucose and lipid profiles were effectively improved in this study. It is evident from our present findings that NaB is protective against the progression of type 2 diabetes.

GATA4 is strongly expressed in endodermal domains, and a molecular analysis indicates that it is crucial for the potentiation of liver gene expression during hepatogenesis [50]. Insulin-sensitive and insulin-resistant states are mediated by tyrosine protein phosphatase nonreceptor type 6 protein (PTPN6), and reducing PTPN6 expression can modulate immune system dysfunction and insulin resistance caused by hyperinsulinemia [51]. In the present study, we found that NaB promoted the expression of GATA4 while inhibiting the expression of PTPN6; these findings suggest that NaB may delay the onset of hepatogenesis; nevertheless, more research is needed to prove this hypothesis.

To further investigate the potential mechanism of NaB on liver function, the intracellular signaling pathway was studied. Previous literature reported that PI3K/Akt signaling pathway participates in insulin resistance, and bile acids can affect cell metabolism and physiological functions by regulating this signaling pathway [52]. As part of the insulin-dependent pathway, insulin receptor substrate-1 (IRS-1) is phosphorylated to begin glucose transport in the liver, and it will further bind with PI3K to activate the subprotein Akt. Activation of Akt was found to inhibit gluconeogenesis and glucose degradation, thereby reducing glucose production. On the other hand, IRS-1 serine phosphorylation was also found to reduce the phosphorylation of tyrosine to suppress insulin signaling, thus suppressing its downstream proteins, such as Akt and PI3K [53]. Based on the findings of this study, NaB administration resulted in the downregulation of p-PI3k, p-Akt, and GSK3. These results suggest that NaB exhibits antidiabetic effects by influencing the PI3K/Akt pathways.

## 5. Conclusions

In conclusion, we found that NaB promotes the metabolism of bile acids by modulating the gut–liver axis and that the PI3K/AKT/GSK3α/β signaling pathway modulates this process. Based on the findings of this study, we also found that both GPR43 expression and mitochondria with membrane potential were reduced under high-insulin and high-LPS stimulation, and NaB treatment ameliorated mitochondrial damage via GPR43. As the metabolite product of intestinal flora, this finding is helpful to understand the pivotal role of dietary supplementation with sodium butyrate in the development of diabetes and further supplies a clue for treating this disease.

## Data Availability

Data will be made available on request.

## Supplementary Materials

The following supporting information can be downloaded at: https://www.mdpi.com/article/10.3390/cimb47090732/s1.

## References

[1] DeFronzo R.A., Ferrannini E., Groop L., Henry R.R., Herman W.H., Holst J.J., Hu F.B., Kahn C.R., Raz I., Shulman G.I. (2015). Type 2 diabetes mellitus. Nat. Rev. Dis. Primers.

[2] Yang M., Wei Y., Liu J., Wang Y., Wang G. (2025). Contributions of Hepatic Insulin Resistance and Islet beta-Cell Dysfunction to the Blood Glucose Spectrum in Newly Diagnosed Type 2 Diabetes Mellitus. Diabetes Metab. J..

[3] Zheng Y., Ley S.H., Hu F.B. (2018). Global aetiology and epidemiology of type 2 diabetes mellitus and its complications. Nat. Rev. Endocrinol..

[4] Mohanty S.K., Singh K., Kumar M., Verma S.S., Srivastava R., Gnyawali S.C., Palakurti R., Sahi A.K., El Masry M.S., Banerjee P. (2024). Vasculogenic skin reprogramming requires TET-mediated gene demethylation in fibroblasts for rescuing impaired perfusion in diabetes. Nat. Commun..

[5] Canfora E.E., Meex R.C.R., Venema K., Blaak E.E. (2019). Gut microbial metabolites in obesity, NAFLD and T2DM. Nat. Rev. Endocrinol..

[6] Danneskiold-Samsoe N.B., Dias de Freitas Queiroz Barros H., Santos R., Bicas J.L., Cazarin C.B.B., Madsen L., Kristiansen K., Pastore G.M., Brix S., Marostica Junior M.R. (2019). Interplay between food and gut microbiota in health and disease. Food Res. Int..

[7] Liu H., Zhang M., Ma Q., Tian B., Nie C., Chen Z., Li J. (2020). Health beneficial effects of resistant starch on diabetes and obesity via regulation of gut microbiota: A review. Food Funct..

[8] Weiss G.A., Hennet T. (2017). Mechanisms and consequences of intestinal dysbiosis. Cell Mol. Life Sci..

[9] Wu J., Yang K., Fan H., Wei M., Xiong Q. (2023). Targeting the gut microbiota and its metabolites for type 2 diabetes mellitus. Front. Endocrinol..

[10] Du L., Li Q., Yi H., Kuang T., Tang Y., Fan G. (2022). Gut microbiota-derived metabolites as key actors in type 2 diabetes mellitus. Biomed. Pharmacother..

[11] Sabatino A., Regolisti G., Cosola C., Gesualdo L., Fiaccadori E. (2017). Intestinal Microbiota in Type 2 Diabetes and Chronic Kidney Disease. Curr. Diabetes Rep..

[12] Jayachandran M., Christudas S., Zheng X., Xu B. (2022). Dietary fiber konjac glucomannan exerts an antidiabetic effect via inhibiting lipid absorption and regulation of PPAR-gamma and gut microbiome. Food Chem..

[13] Salazar J., Angarita L., Morillo V., Navarro C., Martinez M.S., Chacin M., Torres W., Rajotia A., Rojas M., Cano C. (2020). Microbiota and Diabetes Mellitus: Role of Lipid Mediators. Nutrients.

[14] Tong A., Li Z., Liu X., Ge X., Zhao R., Liu B., Zhao L., Zhao C. (2024). Laminaria japonica polysaccharide alleviates type 2 diabetes by regulating the microbiota-gut-liver axis: A multi-omics mechanistic analysis. Int. J. Biol. Macromol..

[15] Mirzaei R., Dehkhodaie E., Bouzari B., Rahimi M., Gholestani A., Hosseini-Fard S.R., Keyvani H., Teimoori A., Karampoor S. (2022). Dual role of microbiota-derived short-chain fatty acids on host and pathogen. Biomed. Pharmacother..

[16] Gao Z., Yin J., Zhang J., Ward R.E., Martin R.J., Lefevre M., Cefalu W.T., Ye J. (2009). Butyrate improves insulin sensitivity and increases energy expenditure in mice. Diabetes.

[17] Zhang W.Q., Zhao T.T., Gui D.K., Gao C.L., Gu J.L., Gan W.J., Huang W., Xu Y., Zhou H., Chen W.N. (2019). Sodium Butyrate Improves Liver Glycogen Metabolism in Type 2 Diabetes Mellitus. J. Agric. Food Chem..

[18] Shapiro H., Kolodziejczyk A.A., Halstuch D., Elinav E. (2018). Bile acids in glucose metabolism in health and disease. J. Exp. Med..

[19] Shao J.W., Ge T.T., Chen S.Z., Wang G., Yang Q., Huang C.H., Xu L.C., Chen Z. (2021). Role of bile acids in liver diseases mediated by the gut microbiome. World J. Gastroenterol..

[20] Chen B., Bai Y., Tong F., Yan J., Zhang R., Zhong Y., Tan H., Ma X. (2023). Glycoursodeoxycholic acid regulates bile acids level and alters gut microbiota and glycolipid metabolism to attenuate diabetes. Gut Microbes.

[21] Xu Y.H., Gao C.L., Guo H.L., Zhang W.Q., Huang W., Tang S.S., Gan W.J., Xu Y., Zhou H., Zhu Q. (2018). Sodium butyrate supplementation ameliorates diabetic inflammation in db/db mice. J. Endocrinol..

[22] European Commission, Directorate-General for Environment (2019). Caring for Animals Aiming for Better Science: Directive 2010/63/EU on Protection of Animals Used for Scientific Purposes: Animal Welfare Bodies and National Committees.

[23] Zhao T., Gu J., Zhang H., Wang Z., Zhang W., Zhao Y., Zheng Y., Zhang W., Zhou H., Zhang G. (2020). Sodium Butyrate-Modulated Mitochondrial Function in High-Insulin Induced HepG2 Cell Dysfunction. Oxid. Med. Cell Longev..

[24] Sayin S.I., Wahlstrom A., Felin J., Jantti S., Marschall H.U., Bamberg K., Angelin B., Hyotylainen T., Oresic M., Backhed F. (2013). Gut microbiota regulates bile acid metabolism by reducing the levels of tauro-beta-muricholic acid, a naturally occurring FXR antagonist. Cell Metab..

[25] Beuling E., Kerkhof I.M., Nicksa G.A., Giuffrida M.J., Haywood J., aan de Kerk D.J., Piaseckyj C.M., Pu W.T., Buchmiller T.L., Dawson P.A. (2010). Conditional Gata4 deletion in mice induces bile acid absorption in the proximal small intestine. Gut.

[26] Wang Z., Li X.L., Hong K.F., Zhao T.T., Dong R.X., Wang W.M., Li Y.T., Zhang G.L., Lin J., Gui D.K. (2021). Total flavonoids of Astragalus Ameliorated Bile Acid Metabolism Dysfunction in Diabetes Mellitus. Evid. Based Complement. Altern. Med..

[27] Li S.Z., Zeng S.L., Liu E.H. (2022). Anti-obesity natural products and gut microbiota. Food Res. Int..

[28] Xia T., Zhang Z., Zhao Y., Kang C., Zhang X., Tian Y., Yu J., Cao H., Wang M. (2022). The anti-diabetic activity of polyphenols-rich vinegar extract in mice via regulating gut microbiota and liver inflammation. Food Chem..

[29] Stachowicz N., Kiersztan A. (2013). The role of gut microbiota in the pathogenesis of obesity and diabetes. Postepy Hig. Med. Dosw..

[30] Qin J., Li Y., Cai Z., Li S., Zhu J., Zhang F., Liang S., Zhang W., Guan Y., Shen D. (2012). A metagenome-wide association study of gut microbiota in type 2 diabetes. Nature.

[31] Slouha E., Rezazadah A., Farahbod K., Gerts A., Clunes L.A., Kollias T.F. (2023). Type-2 Diabetes Mellitus and the Gut Microbiota: Systematic Review. Cureus.

[32] Larsen N., Vogensen F.K., van den Berg F.W., Nielsen D.S., Andreasen A.S., Pedersen B.K., Al-Soud W.A., Sorensen S.J., Hansen L.H., Jakobsen M. (2010). Gut microbiota in human adults with type 2 diabetes differs from non-diabetic adults. PLoS ONE.

[33] Canani R.B., Costanzo M.D., Leone L., Pedata M., Meli R., Calignano A. (2011). Potential beneficial effects of butyrate in intestinal and extraintestinal diseases. World J. Gastroenterol..

[34] Kim C.H., Park J., Kim M. (2014). Gut microbiota-derived short-chain Fatty acids, T cells, and inflammation. Immune Netw..

[35] Van Itallie C.M., Anderson J.M. (2006). Claudins and epithelial paracellular transport. Annu. Rev. Physiol..

[36] Sanchez de Medina F., Romero-Calvo I., Mascaraque C., Martinez-Augustin O. (2014). Intestinal inflammation and mucosal barrier function. Inflamm. Bowel Dis..

[37] Huang W., Man Y., Gao C., Zhou L., Gu J., Xu H., Wan Q., Long Y., Chai L., Xu Y. (2020). Short-Chain Fatty Acids Ameliorate Diabetic Nephropathy via GPR43-Mediated Inhibition of Oxidative Stress and NF-kappaB Signaling. Oxid. Med. Cell Longev..

[38] Pinti M.V., Fink G.K., Hathaway Q.A., Durr A.J., Kunovac A., Hollander J.M. (2019). Mitochondrial dysfunction in type 2 diabetes mellitus: An organ-based analysis. Am. J. Physiol. Endocrinol. Metab..

[39] Schmid A.I., Szendroedi J., Chmelik M., Krssak M., Moser E., Roden M. (2011). Liver ATP synthesis is lower and relates to insulin sensitivity in patients with type 2 diabetes. Diabetes Care.

[40] Ruegsegger G.N., Creo A.L., Cortes T.M., Dasari S., Nair K.S. (2018). Altered mitochondrial function in insulin-deficient and insulin-resistant states. J. Clin. Investig..

[41] Wang Y., Zhou H., Palyha O., Mu J. (2019). Restoration of insulin receptor improves diabetic phenotype in T2DM mice. JCI Insight.

[42] Petersen K.F., Shulman G.I. (2006). Etiology of insulin resistance. Am. J. Med..

[43] Thorens B. (2015). GLUT2, glucose sensing and glucose homeostasis. Diabetologia.

[44] Steiner C., Othman A., Saely C.H., Rein P., Drexel H., von Eckardstein A., Rentsch K.M. (2011). Bile acid metabolites in serum: Intraindividual variation and associations with coronary heart disease, metabolic syndrome and diabetes mellitus. PLoS ONE.

[45] Li T., Francl J.M., Boehme S., Ochoa A., Zhang Y., Klaassen C.D., Erickson S.K., Chiang J.Y. (2012). Glucose and insulin induction of bile acid synthesis: Mechanisms and implication in diabetes and obesity. J. Biol. Chem..

[46] Swanson H.I., Wada T., Xie W., Renga B., Zampella A., Distrutti E., Fiorucci S., Kong B., Thomas A.M., Guo G.L. (2013). Role of nuclear receptors in lipid dysfunction and obesity-related diseases. Drug Metab. Dispos..

[47] Kim Y.J., Jung U.J. (2019). Honokiol Improves Insulin Resistance, Hepatic Steatosis, and Inflammation in Type 2 Diabetic db/db Mice. Int. J. Mol. Sci..

[48] Music M., Dervisevic A., Pepic E., Lepara O., Fajkic A., Ascic-Buturovic B., Tuna E. (2015). Metabolic Syndrome and Serum Liver Enzymes Level at Patients with Type 2 Diabetes Mellitus. Med. Arch..

[49] Khan H.A., Sobki S.H., Khan S.A. (2007). Association between glycaemic control and serum lipids profile in type 2 diabetic patients: HbA1c predicts dyslipidaemia. Clin. Exp. Med..

[50] Watt A.J., Zhao R., Li J., Duncan S.A. (2007). Development of the mammalian liver and ventral pancreas is dependent on GATA4. BMC Dev. Biol..

[51] Marin-Juez R., Jong-Raadsen S., Yang S., Spaink H.P. (2014). Hyperinsulinemia induces insulin resistance and immune suppression via Ptpn6/Shp1 in zebrafish. J. Endocrinol..

[52] Zhang Z., Liu H., Liu J. (2019). Akt activation: A potential strategy to ameliorate insulin resistance. Diabetes Res. Clin. Pract..

[53] Yuan Q., Zhan B., Chang R., Du M., Mao X. (2020). Antidiabetic Effect of Casein Glycomacropeptide Hydrolysates on High-Fat Diet and STZ-Induced Diabetic Mice via Regulating Insulin Signaling in Skeletal Muscle and Modulating Gut Microbiota. Nutrients.

