# Supplementary material for: Sodium Butyrate Ameliorated Bile Acid Metabolism in Diabetes Mellitus by PI3K/AKT Signaling Pathway via the Gut–Liver Axis
Source: Curr Issues Mol Biol. 2025 Sep 9;47(9):732. doi: 10.3390/cimb47090732 (PMC12468112; doi:10.3390/cimb47090732)
Supplement: Supplementary file 1 [file cimb-47-00732-s001.zip › cimb-3821613-supplementary.pdf]

Supplementary Tables

Table S1. List of sequences for primers used in PCR analysis.

Primers name	Forward primers (5′ to 3′)	Reverse primers (5′ to 3′)
FXR	ACATCCCCATCTCTCTGCAC	TGTGAGGGCTGCAAAGGTTT
TGR5	ACTGTCCCTTCTGGCTCTTC	CGGGCCTGGAACTCTGTTAT
ASBT	ACAGGTGCCGAACAGTAG	GATGAGTGGGAAGGTGAA
GAPDH	CTGCGACTTCAACAGCAACT	GAGTTGGGATAGGGCCTCTC

Table S2. List of the top ten differentially expressed proteins

Accession	GeneSymbol	pI	Drug:Model State
sp|A2AS89|SPEB_MOUSE	Agmat	8.0	up
sp|O08553|DPYL2_MOUSE	Dpysl2	6.3	down
tr|Q3UN47|Q3UN47_MOUSE	Endog	9.9	up
sp|O09131|GSTO1_MOUSE	Gsto1	7.5	up
tr|Q9QXK4|Q9QXK4_MOUSE	Cyp3a25	9.2	up
sp|O35381|AN32A_MOUSE	Anp32a	3.7	down
tr|Q8C1X9|Q8C1X9_MOUSE	Anxa3	5.5	down
tr|Q8C2A3|Q8C2A3_MOUSE	Septin7	9.0	down
sp|O55222|ILK_MOUSE	Ilk	8.2	down
sp|O55234|PSB5_MOUSE	Psmb5	7.0	up

The criteria for determining significant differences in expression are: when adj. Pval (Q-value) ⩽ 0.05, fold change (Fold Change) ⩾ 1.2 (up-regulated expression) or fold change (Fold Change) ⩽ 0.833 (down-regulated expression), it is considered Significant changes in expression

Supplementary Figures

Figure S1. The native image of western blot of Figure 8

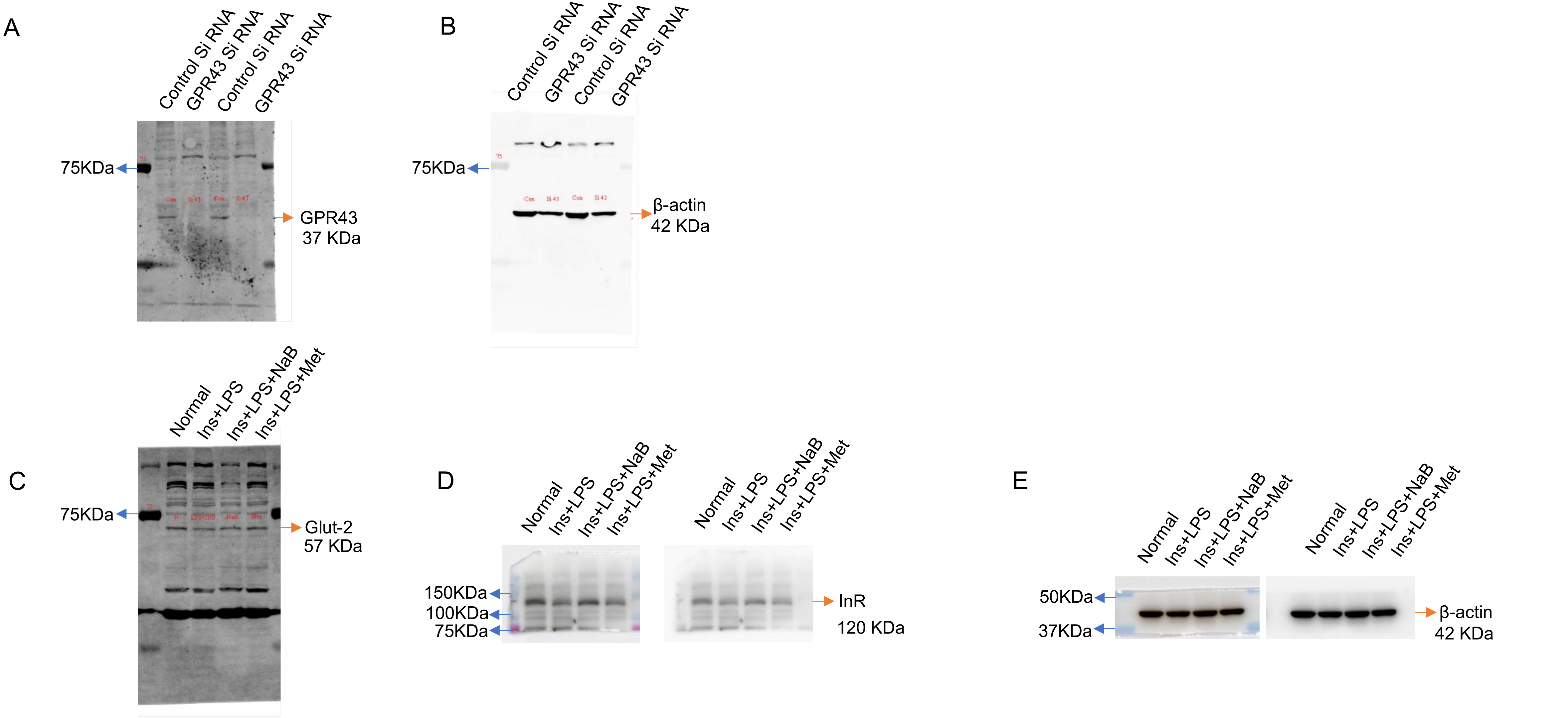

Original images of chemiluminescence exposure of GPR43 (A), β-actin (A) and Glut-2 (C); Original images of fluorescence exposure of insulin receptor (D) and β-actin (E); The blue arrow is the maker, and the orange arrow is the target protein.

Figure S2. The native image of western blot of Figure 9

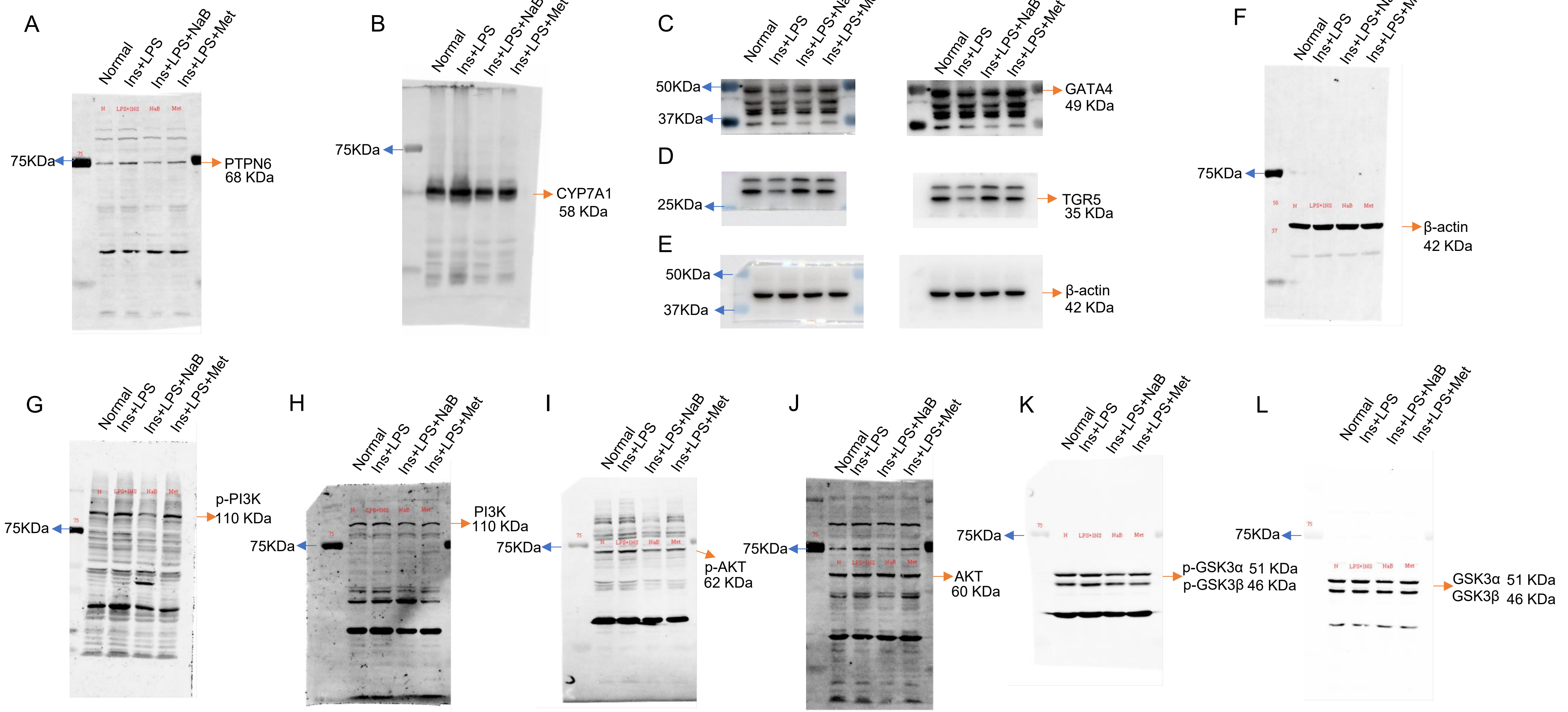

Original images of chemiluminescence exposure of PTPN6 (A), CYP7A1 (B), β-actin (F), p-PI3K (G), PI3K (H), p-AKT (I), AKT (J), p-GSK3α/β (K) and GSK3α/β (L); Original images of fluorescence exposure of GATA4 (C), TGR5 (D) and β-actin (E); The blue arrow is the maker, and the orange arrow is the target protein.

Figure S3-1

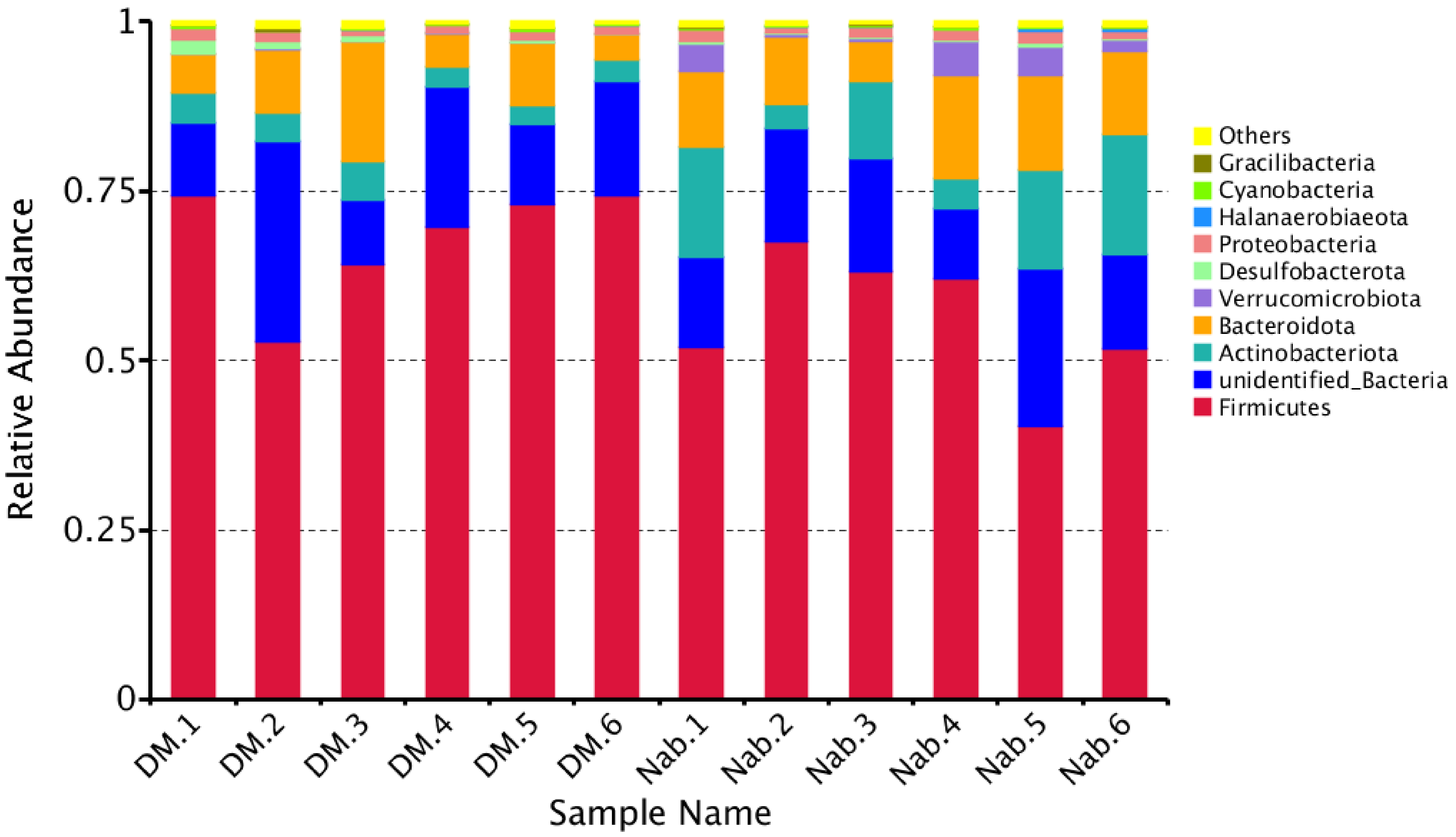

Relative Abundance
1
0.75
0.5
0.25
0
DM.1
DM.2
DM.3
DM.4
DM.5
DM.6
Nab.1
Nab.2
Nab.3
Nab.4
Nab.5
Nab.6
Sample Name
Others
Gracilibacteria
Cyanobacteria
Halanaerobiaeota
Proteobacteria
Desulfobacterota
Verrucomicrobiota
Bacteroidota
Actinobacteriota
unidentified_Bacteria
Firmicutes

Figure S3-2

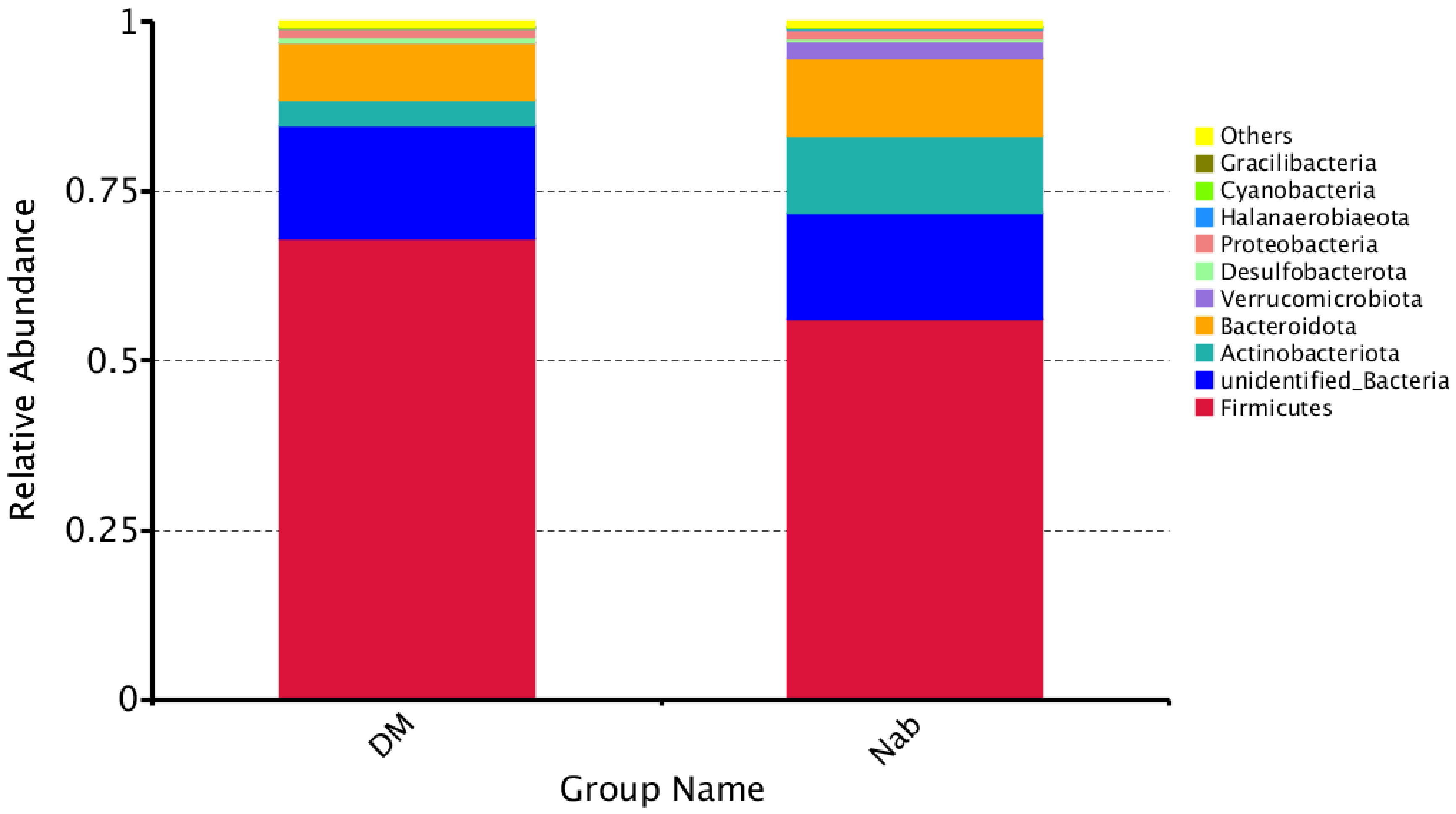
1
0.75
0.5
0.25
0
Relative Abundance
DM
Nab
Group Name
Others
Gracilibacteria
Cyanobacteria
Halanaerobiaeota
Proteobacteria
Desulfobacterota
Verrucomicrobiota
Bacteroidota
Actinobacteriota
unidentified_Bacteria
Firmicutes

Figure S3-3

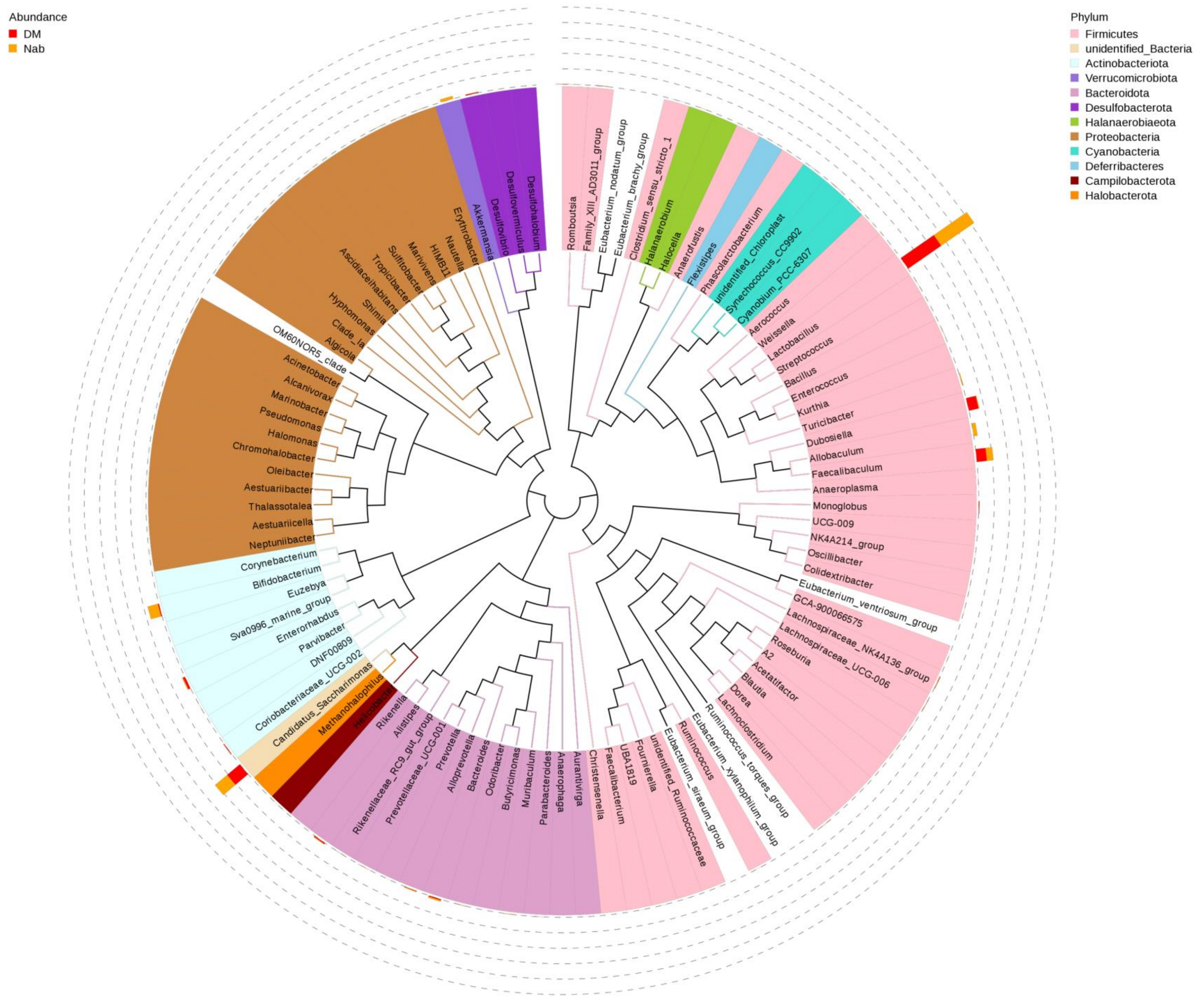
Abundance
DM
Nab
Phylum
Firmicutes
unidentified_Bacteria
Actinobacteriota
Verrucomicrobiota
Bacteroidota
Desulfobacterota
Halanaerobiaeota
Proteobacteria
Cyanobacteria
Deferribacteres
Campilobacterota
Halobacterota
Romboutsia
Family_XIII_AD3011_group
Eubacterium_nodatum_group
Eubacterium_brachy_group
Clostridium_sensu_stricto_1
Halanaerobium
Halocella
Anaerofustis
Flexistipes
Phascolarctobacterium
unidentified_Chloroplast
Synechococcus_CC9902
Cyanobium_PCC-6307
Aerococcus
Weissella
Lactobacillus
Streptococcus
Bacillus
Enterococcus
Kurthia
Turicibacter
Dubosiella
Allobaculum
Faecalibaculum
Anaeroplasma
Monoglobus
UCG-009
NK4A214_group
Oscillibacter
Colidextribacter
Eubacterium_ventriosum_group
GCA-900066575
Lachnospiraceae_NK4A136_group
Lachnospiraceae_UCG-006
Roseburia
A2
Acetatifactor
Blautia
Dorea
Lachnoclostridium
Ruminococcus_torques_group
Eubacterium_xylanophilum_group
Ruminococcus
Eubacterium_siraeum_group
unidentified_Ruminococcaceae
Fournierella
UBA1819
Faecalibacterium
Christensenella
Aurantivirga
Anaerophaga
Parabacteroides
Muribaculum
Butyricimonas
Odoribacter
Bacteroides
Alloprevotella
Prevotella
Prevotellaceae_UCG-001
Rikenellaceae_RC9_gut_group
Alistipes
Rikenella
Helicobacter
Methanohalophilus
Candidatus_Saccharimonas
Coriobacteriaceae_UCG-002
DNF00809
Parvibacter
Enterorhabdus
Sva0996_marine_group
Euzebya
Bifidobacterium
Corynebacterium
Neptuniibacter
Aestuariicella
Thalassotalea
Aestuariibacter
Oleibacter
Chromohalobacter
Halomonas
Pseudomonas
Marinobacter
Alcanivorax
Acinetobacter
OM60NOR5_clade
Algicola
Clade_Ia
Hyphomonas
Shimia
Ascidiaceihabitans
Tropicibacter
Sulfitobacter
Marivivens
HIMB11
Nautella
Erythrobacter
Akkermansia
Desulfovibrio
Desulfovermiculus
Desulfohalobium

Figure S3-4

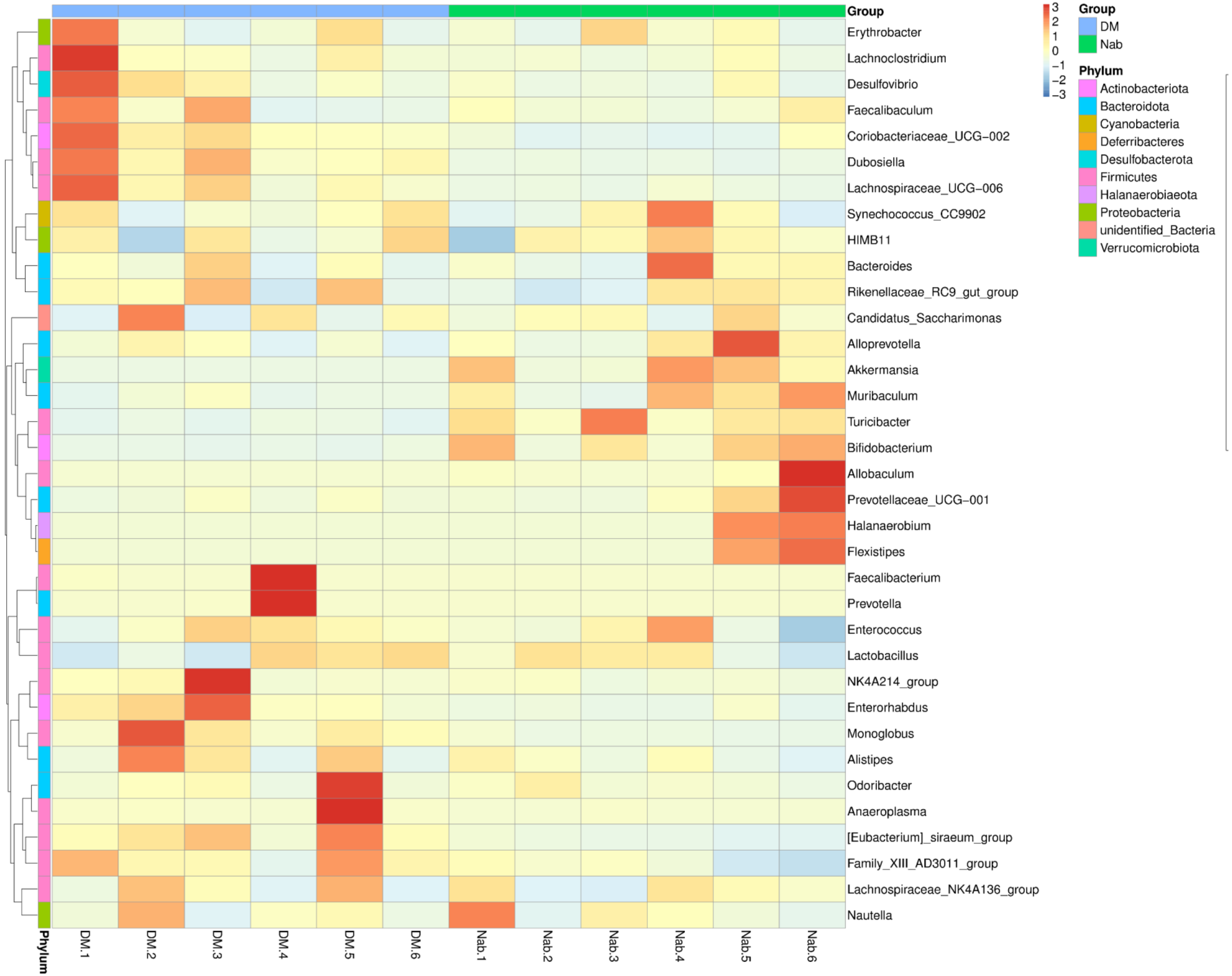
Group
Erythrobacter
Lachnoclostridium
Desulfovibrio
Faecalibaculum
Coriobacteriaceae_UCG-002
Dubosiella
Lachnospiraceae_UCG-006
Synechococcus_CC9902
HIMB11
Bacteroides
Rikenellaceae_RC9_gut_group
Candidatus_Saccharimonas
Alloprevotella
Akkermansia
Muribaculum
Turicibacter
Bifidobacterium
Allobaculum
Prevotellaceae_UCG-001
Halanaerobium
Flexistipes
Faecalibacterium
Prevotella
Enterococcus
Lactobacillus
NK4A214_group
Enterorhabdus
Monoglobus
Alistipes
Odoribacter
Anaeroplasma
[Eubacterium]_siraeum_group
Family_XIII_AD3011_group
Lachnospiraceae_NK4A136_group
Nautella
Phylum
DM.1
DM.2
DM.3
DM.4
DM.5
DM.6
Nab.1
Nab.2
Nab.3
Nab.4
Nab.5
Nab.6
3
2
1
0
−1
−2
−3
Group
DM
Nab
Phylum
Actinobacteriota
Bacteroidota
Cyanobacteria
Deferribacteres
Desulfobacterota
Firmicutes
Halanaerobiaeota
Proteobacteria
unidentified_Bacteria
Verrucomicrobiota

Figure S3-5

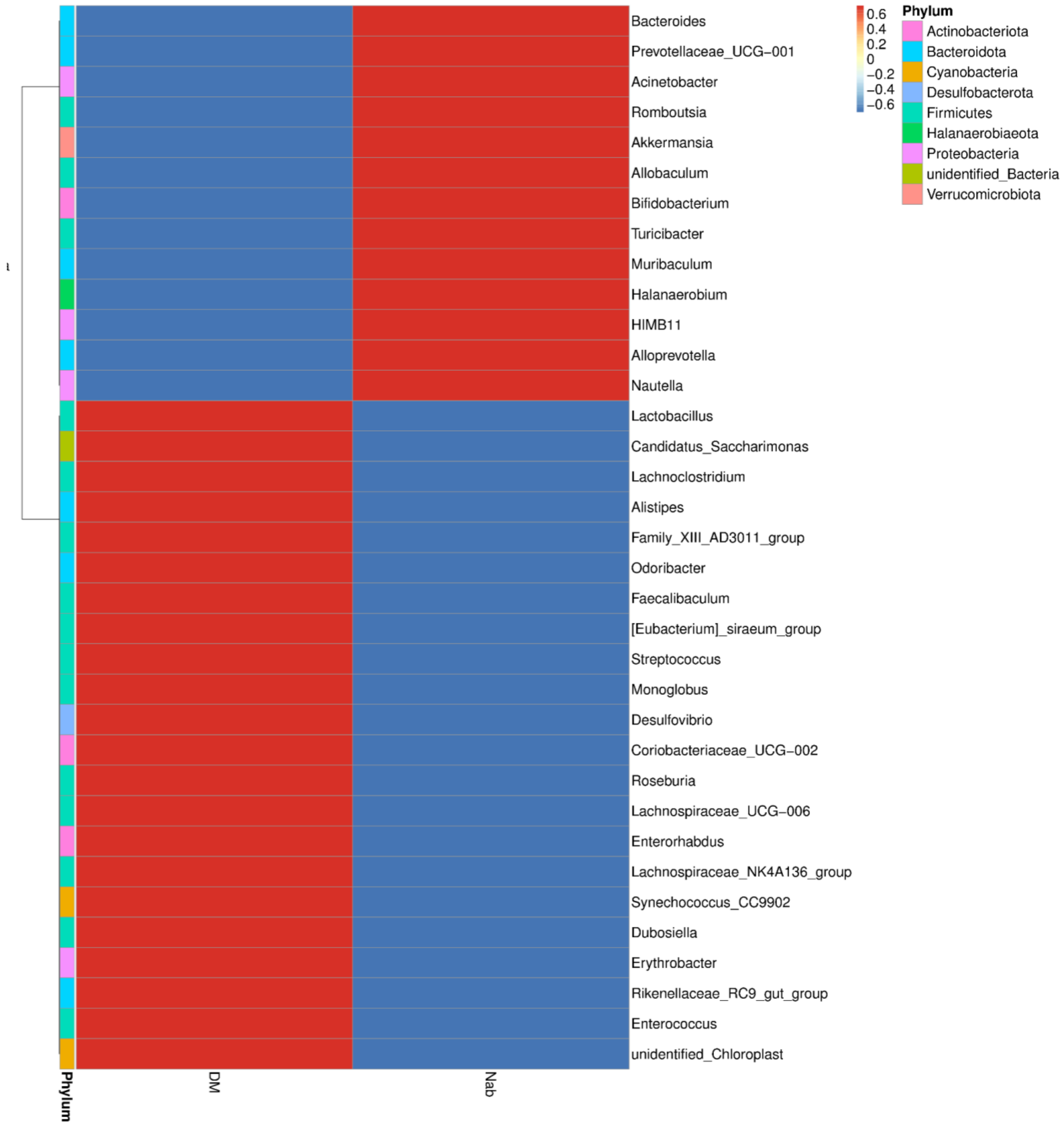
Bacteroides
Prevotellaceae_UCG−001
Acinetobacter
Romboutsia
Akkermansia
Allobaculum
Bifidobacterium
Turicibacter
Muribaculum
Halanaerobium
HIMB11
Alloprevotella
Nautella
Lactobacillus
Candidatus_Saccharimonas
Lachnoclostridium
Alistipes
Family_XIII_AD3011_group
Odoribacter
Faecalibaculum
[Eubacterium]_siraeum_group
Streptococcus
Monoglobus
Desulfovibrio
Coriobacteriaceae_UCG−002
Roseburia
Lachnospiraceae_UCG−006
Enterorhabdus
Lachnospiraceae_NK4A136_group
Synechococcus_CC9902
Dubosiella
Erythrobacter
Rikenellaceae_RC9_gut_group
Enterococcus
unidentified_Chloroplast
Phylum
DM
Nab
0.6
0.4
0.2
0
−0.2
−0.4
−0.6
Phylum
Actinobacteriota
Bacteroidota
Cyanobacteria
Desulfobacterota
Firmicutes
Halanaerobiaeota
Proteobacteria
unidentified_Bacteria
Verrucomicrobiota

Figure S3-6

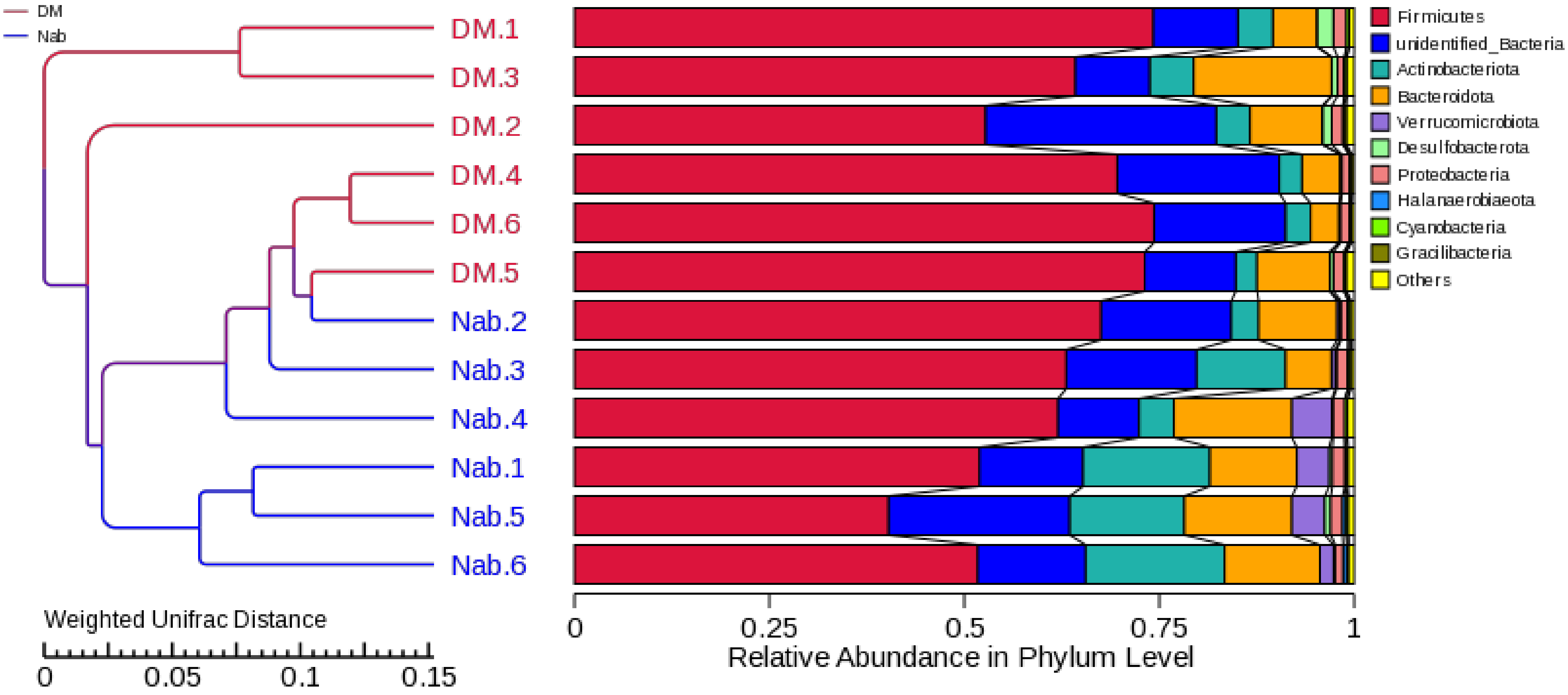

DM
Nab
DM.1
DM.3
DM.2
DM.4
DM.6
DM.5
Nab.2
Nab.3
Nab.4
Nab.1
Nab.5
Nab.6
Weighted Unifrac Distance
0
0.05
0.1
0.15
0
0.25
0.5
0.75
1
Relative Abundance in Phylum Level
Firmicutes
unidentified_Bacteria
Actinobacteriota
Bacteroidota
Verrucomicrobiota
Desulfobacterota
Proteobacteria
Halanaerobiaeota
Cyanobacteria
Gracilibacteria
Others

Figure S3-7

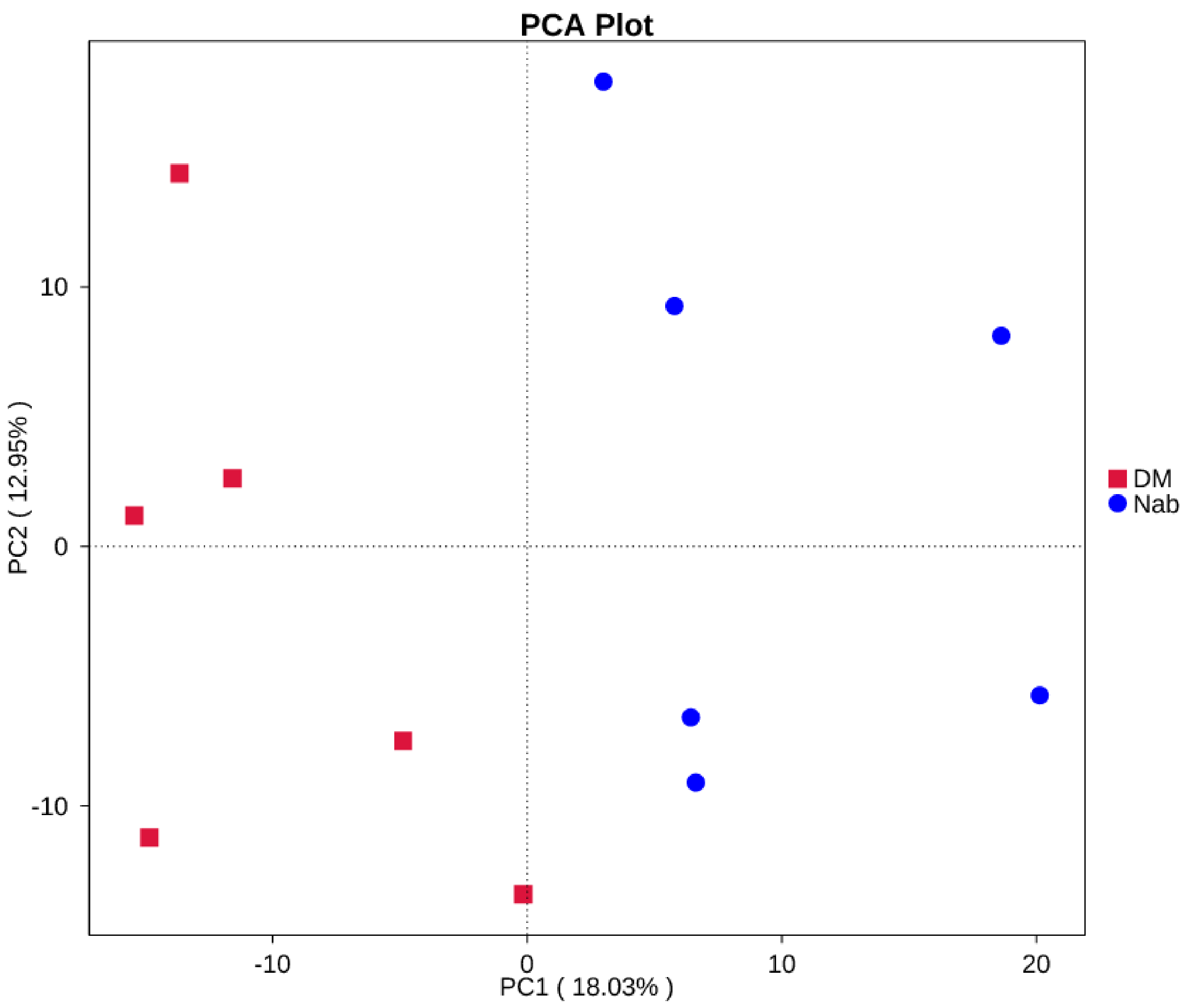

Figure S3-8

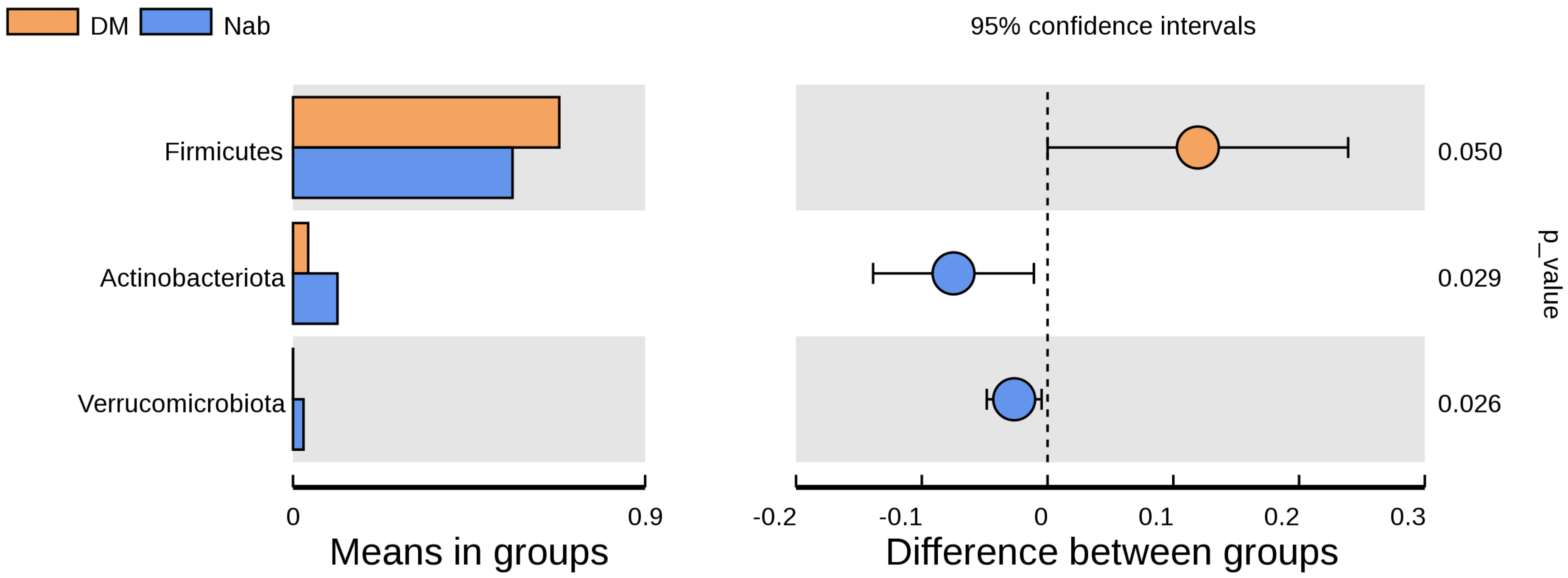

DM
Nab
95% confidence intervals
Firmicutes
Actinobacteriota
Verrucomicrobiota
0.050
0.029
0.026
p_value
0
0.9
Means in groups
-0.2
-0.1
0
0.1
0.2
0.3
Difference between groups
